# EnDecon: cell type deconvolution of spatially resolved transcriptomics data via ensemble learning

**DOI:** 10.1093/bioinformatics/btac825

**Published:** 2022-12-22

**Authors:** Jia-Juan Tu, Hui-Sheng Li, Hong Yan, Xiao-Fei Zhang

**Affiliations:** Centre for Intelligent Multidimensional Data Analysis, Hong Kong Science Park, Hong Kong 999077, China; Centre for Intelligent Multidimensional Data Analysis, Hong Kong Science Park, Hong Kong 999077, China; Department of Statistics, School of Mathematics and Statistics & Hubei Key Laboratory of Mathematical Sciences, Central China Normal University, Wuhan 430079, China; Centre for Intelligent Multidimensional Data Analysis, Hong Kong Science Park, Hong Kong 999077, China; Department of Electrical Engineering, City University of Hong Kong, Hong Kong 999077, China; Department of Statistics, School of Mathematics and Statistics & Hubei Key Laboratory of Mathematical Sciences, Central China Normal University, Wuhan 430079, China

## Abstract

**Motivation:**

Spatially resolved gene expression profiles are the key to exploring the cell type spatial distributions and understanding the architecture of tissues. Many spatially resolved transcriptomics (SRT) techniques do not provide single-cell resolutions, but they measure gene expression profiles on captured locations (spots) instead, which are mixtures of potentially heterogeneous cell types. Currently, several cell-type deconvolution methods have been proposed to deconvolute SRT data. Due to the different model strategies of these methods, their deconvolution results also vary.

**Results:**

Leveraging the strengths of multiple deconvolution methods, we introduce a new weighted ensemble learning deconvolution method, EnDecon, to predict cell-type compositions on SRT data in this work. EnDecon integrates multiple base deconvolution results using a weighted optimization model to generate a more accurate result. Simulation studies demonstrate that EnDecon outperforms the competing methods and the learned weights assigned to base deconvolution methods have high positive correlations with the performances of these base methods. Applied to real datasets from different spatial techniques, EnDecon identifies multiple cell types on spots, localizes these cell types to specific spatial regions and distinguishes distinct spatial colocalization and enrichment patterns, providing valuable insights into spatial heterogeneity and regionalization of tissues.

**Availability and implementation:**

The source code is available at https://github.com/Zhangxf-ccnu/EnDecon.

**Supplementary information:**

Supplementary data are available at *Bioinformatics* online.

## 1 Introduction

Spatially resolved transcriptomics (SRT) technologies perform gene expression profiling on capture tissue locations (spots) while maintaining spatial localization information of spots (Asp *et al.*, 2020; Burgess, 2019; Marx, 2021). Spatially resolved gene expression profiles provide an opportunity to characterize cellular heterogeneity in the spatial context and investigate the architectures of the tissues (Andersson *et al.*, 2021; Burgess, 2019; Dries *et al.*, 2021; Eng *et al.*, 2019; Moses *et al.*, 2022; Pham *et al.*, 2020; Zhang *et al.*, 2021). However, despite the rapid development of SRT, many SRT technologies lack single-cell resolutions, such as the spatial transcriptomics (ST) technique (Ståhl *et al.*, 2016) and the commercialized 10× Genomics Visium system. Each detected spot is generally a mixture of multiple homos or heterogeneous cell types, which may make it difficult to explore the spatial distribution of cell types in complex tissues. Compared with the SRT technologies, single-cell RNA-sequencing (scRNA-seq) technologies enable quantifying transcriptome profiling at the single-cell level, while cells’ spatial localization information is lost during the process of cell isolation (Abdelaal *et al.*, 2020). To explore the spatial cellular heterogeneity, it is reasonable to leverage cell type profiles learned from scRAN-seq data to decompose mixture cells within each spot of SRT data.

In the context of bulk RNA-seq data, which measures the average gene expression level of thousands of cells within a sample, several deconvolution methods have been proposed to infer cell type abundances for each sample, such as DeconRNASeq (Gong *et al.*, 2013), DWLS (Tsoucas *et al.*, 2019), MuSiC (Wang *et al.*, 2019) and SCDC (Dong *et al.*, 2021a). By treating one spot as a bulk sample, the deconvolution methods designed for bulk RNA-seq data could be applied directly to SRT data (Avila Cobos *et al.*, 2020; Sturm *et al.*, 2019). However, compared with bulk RNA-seq data, SRT data contain a lower quantity of cells within each capture spot. For example, the 10× Genomics Visium platform treats an area of 55 μm as a spot, which contains around 5–10 cells. As limited cells are contained on each spot, spatially resolved gene expression profiles are sparser and have lower signals than bulk gene expression profiles (Danaher *et al.*, 2022). On the other hand, SRT data could obtain the spatial localization information of captured spots, providing the opportunity to explore the cell-type spatial distributions in a spatial context. Hence, existing methods developed for deconvoluting bulk RNA-seq data may produce misleading results if one applies them directly to SRT data (Dong *et al.*, 2021b). To accommodate the special characteristics of SRT data, several cell-type deconvolution methods have been developed, such as CARD (Ma *et al.*, 2022), Cell2location (Kleshchevnikov *et al.*, 2022), DestVI (Lopez *et al.*, 2022), RCTD (Cable *et al.*, 2021), STdeconvolve (Miller *et al.*, 2022), Stereoscope (Andersson *et al.*, 2020), SpatialDWLS (Dong *et al.*, 2021b) and SPOTlight (Elosua Bayes *et al.*, 2021).

The deconvolution methods designed for bulk RNA-seq data and SRT data are often based on different model assumptions and strategies (Supplementary Table S1). Each deconvolution method has its own strengths and limitations (Avila Cobos *et al.*, 2020; Chen *et al.*, 2022; Li *et al.*, 2022a). Besides, the deconvolution methods highly depend on the reference scRNA-seq data (Sun *et al.*, 2022) and the complexity of the underlying tissue (Kleshchevnikov *et al.*, 2022). The deconvolution results produced by different methods may be quite different from each other. Thus, it is difficult to choose the best deconvolution method for new SRT data in reality. In machine learning, the weighted average ensemble method is widely used to obtain better performance by weighted averaging of the results from multiple base methods (Dong *et al.*, 2021a; Li *et al.*, 2022b; Zhang *et al.*, 2019). Therefore, a weighted average ensemble method can also be developed to deconvolute the SRT data. However, how to determine the weights assigned to the base deconvolution methods is a key issue. If each method is assigned the same weight, it is possible to produce a poor ensemble result due to the influence of the poorly performing methods.

**Fig. 1.**
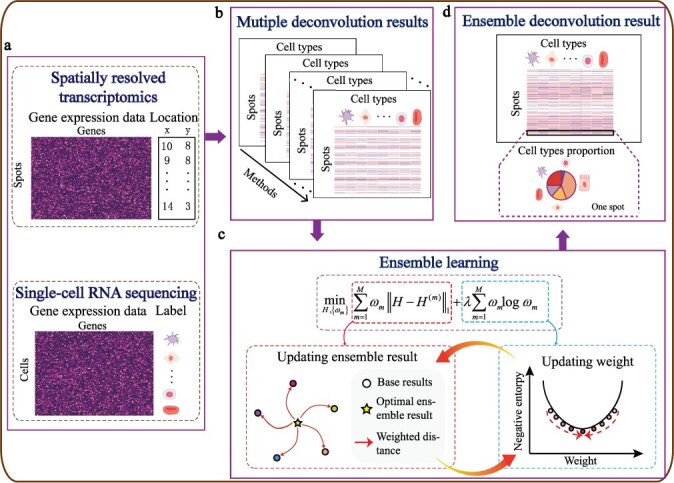
Overview of EnDecon. (**a**) EnDecon takes spatially resolved transcriptomics data with spot localizations and annotated reference scRNA-seq dataset as input. Spatially resolved gene expression data is performed for deconvolution to obtain cell type compositions on each capture spot. The scRNA-seq dataset consisting of a gene expression matrix and corresponding cell label information serves as reference cell type-specific gene expression data. (**b**) Multiple deconvolution methods are run individually to obtain multiple base deconvolution results on the SRT data. (**c**) EnDecon uses a new optimization model to integrate these base results. The optimal ensemble result is obtained by alternatively updating the ensemble result and the weights of base results. (**d**) EnDecon predicts an ensemble deconvolution result based on multiple based deconvolution results from the SRT data. Each spot is composed of varying cell types with different proportions

In this study, we propose an ensemble learning-based cell-type deconvolution method (Fig. 1), called EnDecon, to estimate cell-type abundances within spots by borrowing strengths from existing cell-type deconvolution methods. EnDecon utilizes an optimization strategy for the combination of the base deconvolution results from 14 individual methods to produce a consistent and accurate deconvolution result. It achieves the optimal solution by alternatively updating the ensemble result, which is produced based on a weighted median of the base deconvolution results, and the weights of base results, which are evaluated based on their Manhattan distances to the corresponding ensemble results. Simulation results show the advantages of EnDecon over base deconvolution methods and a baseline ensemble learning method in terms of inferred cell type proportions on spots. Furthermore, strong positive correlations are observed between the weights learned by EnDecon and the performance of base deconvolution methods. Comprehensive experiments on four real SRT data show that EnDecon has advantages in predicting the cell type proportions on spots in terms of tissue region segmentation, concordance analysis of the spatial distribution of cell types and their marker genes, region-based cell type enrichment analysis and cell type colocalization analysis.

## 2 Materials and methods

### 2.1 Overview of EnDecon

Given observed SRT data and a coupled reference scRNA-seq gene expression dataset with pre-defined cell type information, EnDecon leverages the ensemble learning approach to integrate the results from multiple base deconvolution methods. The overview of EnDecon is presented in Figure 1. EnDecon includes two main steps (i) running each base deconvolution method individually to obtain the base cell type deconvolution results and (ii) integrating these base deconvolution results into a better deconvolution result using a new proposed ensemble strategy. EnDecon obtains the ensemble result by alternatively updating the ensemble result as a weighted median of the base results, and the weights of base results based on their distances to their corresponding ensemble results.

### 2.2 Implementation of the individual deconvolution methods

The current implementation of EnDecon combines 14 state-of-the-art cell-type deconvolution methods (consisting of methods designed for both bulk RNA-seq and scRNA-seq datasets): Conditional Auto Regressive-based Deconvolution (CARD) (Ma *et al.*, 2022), Cell2location (Kleshchevnikov *et al.*, 2022), DeconRNASeq (Gong *et al.*, 2013), DestVI (Lopez *et al.*, 2022), Dampened Weighted Least Squares (DWLS) (Tsoucas *et al.*, 2019), v-support vector regression (SVR) (Tsoucas *et al.*, 2019), MUlti-Subject SIngle Cell deconvolution (MuSiC) (Wang *et al.*, 2019), Robust Cell Type Decomposition (RCTD) (Cable *et al.*, 2021), SCDC (Dong *et al.*, 2021a), SpatialDWLS (Dong *et al.*, 2021b), SPOTlight (Elosua Bayes *et al.*, 2021), STdeconvolve (Miller *et al.*, 2022) and Stereoscope (Andersson *et al.*, 2020). The main features of these base deconvolution methods are summarized in Supplementary Table S1. We run these base deconvolution methods using the packages provided by the authors and following the corresponding guidelines. For details, please refer to Supplementary Section S3.1.

### 2.3 Integrating deconvolution results generated by individual deconvolution methods

After applying the 14 base deconvolution methods mentioned above, we can obtain 14 base deconvolution results $H^{\left(m\right)}\in R^{N\times K}$, where *N* represents the number of spots, *K* represents the number of cell types, and *m* represents the *m*th base deconvolution method for m=1,…,M (*M *=* *14 by default). The goal of this study is to learn an ensemble cell type deconvolution result *H* by integrating the results generated by the 14 base deconvolution methods.

Due to the variation of base deconvolution results, integrating multiple base deconvolution results may help to learn a better ensemble deconvolution result. A widely used ensemble learning strategy is to treat each base deconvolution result equally and use their average as the ensemble result, which we refer to as EnDecon_mean in this article. However, the accuracy of different deconvolution results may be different. If we treat these base deconvolution results equally, the poor performance results may lead to biased deconvolution results. Instead of using an average ensemble strategy, we propose the following optimization model to integrate these base results (called as EnDecon),

(1) $$\begin{array}{c} \underset{H,\left\{\omega_{m}\right\}}{\min}\;\sum_{m=1}^{M}\omega_{m}{\left\|H-H^{\left(m\right)}\right\|}_{1}+\lambda \sum_{m=1}^{M}\omega_{m}\;\text{log}\;\omega_{m}\\ \text{subject}\;\text{to}\;\sum_{m=1}^{M}\omega_{m}=1,\;\omega_{m}\geq 0. \end{array},$$

where the first term measures the total weighted Manhattan distance between the ensemble deconvolution result *H* and base deconvolution results $H^{\left(m\right)},\;{\left\|.\right\|}_{1}$ represents the *L*_1_ norm of a matrix, and *ω_m_* represents the weight assigned to the *m*th base deconvolution result. By minimizing this term with respect to *H*, we can learn a consensus cell-type abundance matrix. The second term is a negative entropy regularization of weights, which is used to prevent the ensemble deconvolution result from overfitting to a certain base deconvolution result. By minimizing the first term and the regularization term with respect to *ω*, we can learn weights assigned to the base deconvolution results. If the value of weight *ω_m_* is large, the ensemble result is encouraged to be close to the corresponding base deconvolution result $H^{\left(m\right)}$. Otherwise, the ensemble result may be apart from this deconvolution result. The parameter *λ* is a tuning parameter to control the strength of regularization. After obtaining the estimates of model parameters, EnDecon can obtain an ensemble deconvolution result *H* by assigning appropriate weights *ω_m_* to different base deconvolution results $H^{\left(m\right)}$.

### 2.4 Optimization algorithm

There are two sets of model parameters, *H* and $\left\{\omega_{m}\right\}$, to estimate in Equation (1). We develop a coordinate descent algorithm to solve the optimization problem, in which we iteratively update one parameter while keeping the other constant.

We first estimate *H* while holding $\left\{\omega_{m}\right\}$ fixed. When we calculate *H*, the optimization problem can be rewritten as follows:

(2) $$\begin{array}{c} \underset{H}{\min}\sum_{m=1}^{M}\omega_{m}{\left\|H-H^{\left(m\right)}\right\|}_{1}. \end{array}$$

The above optimization problem is a classic post office location problem, for which the weighted median would be the solution (Clarkson, 1985; Fletcher *et al.*, 2009). The function *weighted.median* in R package *spatstat.geom* is used to solve Equation (2).

We then compute $\left\{\omega_{m}\right\}$ while keeping model parameter *H* fixed and rewrite the optimization problem in Equation (1) as follows:

(3) $$\begin{array}{c} \underset{\left\{\omega_{m}\right\}}{\min}\;\sum_{m=1}^{M}\omega_{m}{\left\|H-H^{\left(m\right)}\right\|}_{1}+\lambda \sum_{m=1}^{M}\omega_{m}\;\text{log}\;\omega_{m}\\ \text{subject}\;\text{to}\;\sum_{m=1}^{M}\omega_{m}=1,\;\omega_{m}\geq 0. \end{array}$$

According to the method of Lagrange multipliers, the above optimization problem has a closed-form solution for each *ω_m_*:

(4) $$\omega_{m}=\frac{e^{-\frac{1}{\lambda }{\left\|H-H^{\left(m\right)}\right\|}_{1}}}{\sum_{m=1}^{M}e^{-\frac{1}{\lambda }{\left\|H-H^{\left(m\right)}\right\|}_{1}}}.$$

For the coordinate descent algorithm, the iteration process is conducted until the relative change of the objective function in Equation (1) is less than $10^{-5}$. As can be seen from Equation (2), the ensemble result will be a weighted median of base deconvolution results. From Equation (4), the weights assigned to base deconvolution results are associated with their distances from the ensemble result. We will assess the accuracy of the ensemble result and the rationality of the weights learned by EnDecon in the following experiments. The complete procedure of EnDecon is summarized in Supplementary Section S3.2.

In Equation (1), the tuning parameter *λ* is used to control the strength of entropy regularization and needs to be predetermined before estimating *H* and $\left\{\omega_{m}\right\}$. In this work, we set its value empirically to the median of *L*_1_ norm distances between the EnDecon_mean result $\frac{1}{M}\sum_{m=1}^{M}H^{\left(m\right)}$ and these base deconvolution results $\left\{H^{\left(m\right)}\right\}$:

(5) $$\lambda =\mathrm{median}{\left\{{\left\|\frac{1}{M}\sum_{m=1}^{M}H^{\left(m\right)}-H^{\left(m\right)}\right\|}_{1}\right\}}_{m=1}^{M}.$$

## 3 Simulation study

### 3.1 Data generation

To test the performance of different methods, we use single-cell resolution gene expression data to construct spot-based gene expression data and generate corresponding cell type components within spots. Here, we generate simulation data in three different scenarios based on different settings. Specifically, the SRT and scRNA-seq datasets are generated from the same technology in Scenario 1. We conduct two experiments based on data from two different tissues respectively: pancreas [Baron: scRNA-seq data from inDrop (Baron *et al.*, 2016) and ovarian cancer (Ovarian cancer: scRNA-seq data from 10× Genomic]. In Scenario 2, in order to show how batch effects between different techniques affect the deconvolution results, we use the Baron dataset used in Scenario 1 to generate SRT dataset and datasets from other three techniques [e.g. Muraro: CEL-Seq2 (Muraro *et al.*, 2016), Segerstolpe: SMART-Seq2 (Segerstolpe *et al.*, 2016) and Wang: SMARTer (Wang *et al.*, 2016)] as references. In Scenario 3, a real single-cell resolution SRT data from the mouse visual cortex generated by STARmap (Wang *et al.*, 2018) is used to mimic the SRT data, and a scRNA-seq dataset from Smart-seq (Tasic *et al.*, 2018) is used as a reference. Details are provided in Supplementary Section S3.3.1.

### 3.2 Benchmarking different deconvolution methods

We compare the performance of our EnDecon with each base deconvolution method on different simulated SRT data. All base deconvolution methods are run with the recommended default parameters. To demonstrate the advantages of the new ensemble strategy used in EnDecon, we also compare EnDecon with a baseline ensemble method (EnDecon_mean), taking the mean of different base deconvolution results as the ensemble result. Following previous studies (Elosua Bayes *et al.*, 2021; Wang *et al.*, 2019), we quantify the deconvolution performance by computing the Pearson correlation coefficient (PCC), root means square error (RMSE) and *Jensen—Shannon* Divergence distance (JSD) between the predicted and the ground truth cell type compositions within each spot. We measure the rationality of the weights learned by EnDecon by calculating Pearson correlation coefficient (*τ*) and Spearman correlation coefficient (*ρ*) between the learned weights and PCC scores of base methods. Multiple simulation replicates are performed in Scenarios 1 and 2, with a default of 10 times, to capture data variation and assess the robustness of each method. In Scenario 3, we do one replicate as in the previous studies (Cable *et al.*, 2021; Elosua Bayes *et al.*, 2021).

**Fig. 2.**
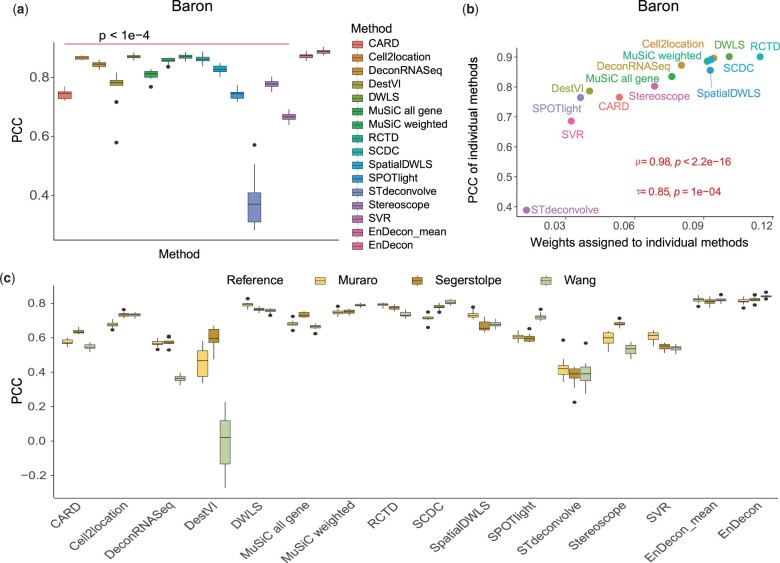
Analysis of the simulation SRT data. (**a**) The performance of the deconvolution methods on simulated pancreas data in Scenario 1. A two-sided *t*-test is performed to assess statistical differences between the results of compared methods and EnDecon. (**b**) The dotplot shows the correlation between the weights inferred by EnDecon and the PCC scores of corresponding base deconvolution methods on simulation data in pancreas data. The Pearson correlation coefficient (*τ*) and Spearman correlation coefficient (*ρ*) between the learned weights and PCC scores of base methods, and the corresponding *P*-values (from a one-sided *t*-test) are provided. (**c**) The performance of the deconvolution methods with reference data from different techniques in Scenario 2

On the pancreas dataset in Scenario 1, EnDecon outperforms all base deconvolution methods (median PCC 0.923), with 1.954%, 2.055% and 2.538% improvements in terms of PCC compared with those of the top three base deconvolution methods, such as RCTD (median 0.905), DWLS (median 0.904) and Cell2location (median 0.900) (Fig. 2a). The statistical differences between the pairwise base deconvolution results and the result of EnDecon are significant. By computing the RMSE between the predicted cell type proportions and the ground truth, EnDecon induces the smallest error (Supplementary Fig. S1a). The calculated JSD indicates that the value of EnDecon (median 0.031) is closer to 0, signifying a higher similarity between the inferred cell type proportions and the ground truth (Supplementary Fig. S1b). In terms of JSD, EnDecon outperforms all base deconvolution methods with 22.153% improvement compared with the top one base method DWLS (median 0.040). Compared with the baseline ensemble method, EnDecon_mean, the performance of EnDecon significantly improves by 1.613%, 5.973% and 111.503% in terms of these three metrics (*t*-test: *P-*value < 0.05 for PCC scores; Diebold–Mariano test: *P-*value <2.2e−16 for RMSE scores; Kolmogorov–Smirnov test: *P-*value <2.2e−16 for JSD scores), respectively. In addition, the weights assigned to base deconvolution methods by EnDecon show a strong and significant positive correlation with the corresponding PCC (median value of each method) (Fig. 2b). EnDecon assigns larger weights to the base deconvolution methods that have higher accuracies and assigns smaller weights to the ones that have poor performance. In line with performance on pancreas data, EnDecon shows the best performance and the learned weights by EnDecon appear positive correlation with PCC scores of the base deconvolution method on ovarian cancer data (Supplementary Figs S2 and S3). These results demonstrate that EnDecon can effectively combine the base methods by automatically assigning larger weights to methods with higher performance.

Then, we use data generated from different platforms in Scenario 2 to check the robustness of EnDecon. We find that the performance of different methods may vary slightly depending on the reference datasets (Fig. 2c and Supplementary Fig. S4). EnDecon provides stable and accurate, either the best or close to the best, results in terms of all three evaluation metrics in most cases (Fig. 2c and Supplementary Fig. S4). With existing batch effects between SRT and reference data, the cell type deconvolution results inferred by some base deconvolution methods such as DestVI and STdeconvolve may depart away from the ground truth. The corresponding weights estimated by EnDecon for these methods are also relatively small (Supplementary Fig. S5). Overall, EnDecon provides comparable and stable results compared to all compared deconvolution methods.

In Scenario 3, we evaluate the performance of EnDecon on more realistic simulated data generated from single-cell resolution SRT data (Supplementary Fig. S6). In terms of PCC, RMSE and JSD, EnDecon is still the top-performing method regards predicting the cell type proportions and shows positive correlation between learned weights and the performance of base deconvolution methods (Supplementary Figs S7 and S8). By visualizing predicted cell type proportions, we find EnDecon accurately localizes L4 excitatory neurons to the specific layer of L4 (Supplementary Fig. S9). In contrast, CARD and ‘MuSiC all gene’ do not capture the expected spatial localization. When integrating all base deconvolution results, EnDecon assigns the smaller weights to them (Supplementary Fig. S8). The excellent performance of EnDecon demonstrates the advantage of our weighted ensemble learning strategy.

Since the sample size obtained may vary for different cell types, we examine the effect of the sample size of the reference scRNA-seq data on the performance of our method (Supplementary Section S3.3.2). The simulation results also show that our method works better than other compared methods. The comparison of running time between different methods on the simulated data is presented in Supplementary Section S3.3.3.

## 4 Evaluating performance using real SRT data

We apply EnDecon to two mouse brain SRT data from 10× Visium protocol and two cancer SRT data [pancreatic ductal adenocarcinoma (PDAC) and breast cancers] from ST protocol to chart spatial cellular heterogeneity (Supplementary Section S3.4.1). First, an adult mouse brain SRT dataset, for which the matched immunofluorescence (IF) staining images for cell-type marker proteins are available, is used to quantitatively evaluate the performance of different deconvolution methods. We then present four applications of EnDecon on the other three datasets: (i) visualization analysis of cell type spatial distribution; (ii) concordance analysis of the spatial distribution of cell types and their corresponding marker genes; (iii) cell type colocalization analysis (Supplementary Section S3.4.2); and (iv) cell type regional enrichment analysis (Supplementary Section S3.4.2). The results on an adult mouse brain and two cancer SRT data are presented as follows and the results on another mouse cortex SRT data are provided in Supplementary Section S3.4.3 due to limited space here.

### 4.1 Evaluation of the performance on an adult mouse brain SRT dataset

**Fig. 3.**
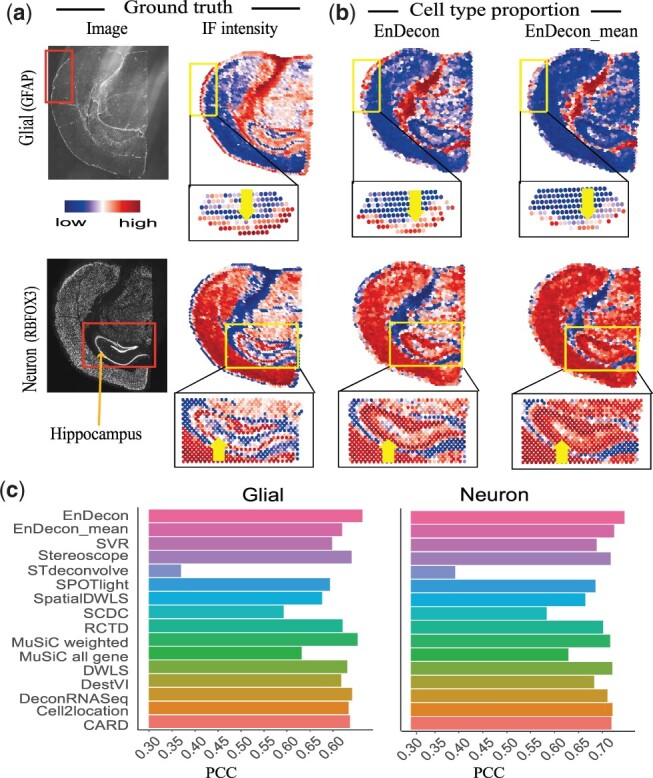
Analysis of the adult mouse brain SRT data (coronal section 2). (**a**) Top, the raw immunofluorescence (IF) images of the adult mouse brain tissue section show the glial (marker protein: GFAP) and neuronal (marker protein: RBFOX3) cells intensity information. Bottom, the spatial distribution of the rescaled intensity values for glial and neuronal cells are matched to each corresponding spot’s spatial location for SRT data. (**b**) Visualization of deconvolution results of EnDecon and EnDecon_mean. Each scatter represents a spot in SRT data. Arrows highlight the strong instance information, where there is a significant difference in cell type proportions predicted by EnDecon and EnDecon_mean. Note that for the convenience of typesetting, when zooming in and visualizing glial cells, we rotate the image 90° counterclockwise. (**c**) Barplot represents PCC scores between the ground truth (intensity values of corresponding glial and neuronal cells) and cell type proportions inferred by different methods

The lack of ground truth for the cell type composition of spots in ST or 10× Visium experiments makes evaluation using real data challenging (Chen *et al.*, 2022; Lopez *et al.*, 2022; Zubair *et al.*, 2022). To compare the performance of EnDecon with other methods, we conduct an experiment following (Zubair *et al.*, 2022). We use a dataset that measures gene expression in an adult mouse brain using the 10× Genomics Visium platform and performs immunofluorescent (IF) staining on the reverse side of tissue sections for two proteins (GFAP and RBFOX3), which are protein markers specific to glial and neurons cells, respectively (Fig. 3a). In a similar way to Zubair *et al.* (2022), we use the estimated spot-level intensity of two marker proteins as ground truth for each matched spot (Fig. 3a). For details, please refer to Supplementary Section 3.4.1.

For each deconvolution method, we predict cell type compositions within spots by leveraging cell type information from the reference scRNA-seq dataset and compute the abundance of glial and neuron cell types for each spot. Cell type abundance inferred by EnDecon can accurately depict these two structures derived from IF image by visual inspection (Fig. 3b), while the results of some compared methods (e.g. ‘MuSiC all gene’, SCDC and STdeconvolve) are not consistent with the expected structures (Supplementary Figs S10 and S11). Compared with EnDecon_mean, EnDecon can correctly capture glial cells outside mouse brain tissue (highlighted by arrows) that are incorrectly predicted as neuron cells by EnDecon_mean, and the neuron cells in the hippocampal region (highlighted arrows) that are incorrectly predicted as glial cells by EnDecon_mean (Fig. 3b).

To quantitatively compare the performance of different deconvolution methods, we calculate the PCC between cell type proportions estimated by different deconvolution methods and IF-derived intensities of corresponding marker proteins for each cell type (Zubair *et al.*, 2022). EnDecon obtains the largest PCC values (0.745 for glial cells and 0.764 for neuron cells) (Fig. 3c). It achieves improvements of 2.762%, 3.223% and 3.310% compared with those of the top three methods EnDecon_mean (0.724), Cell2location (0.721) and DWLS (0.720) for glial cells, and 1.427%, 2.945% and 3.048% compared with those of the top three methods ‘MuSiC weighted’ (0.753), DeconRNASeq (0.741) and Stereoscope (0.740) for neuron cells. The weights assigned to base deconvolution methods by EnDecon are positively correlated to their performance (Supplementary Fig. S12). Overall, EnDecon is competitive with existing deconvolution methods on real SRT data, and the weights assigned to the base methods by EnDecon may provide a clue about their performance when ground truth is not available.

### 4.2 Charting spatial heterogeneity in PDAC

**Fig. 4.**
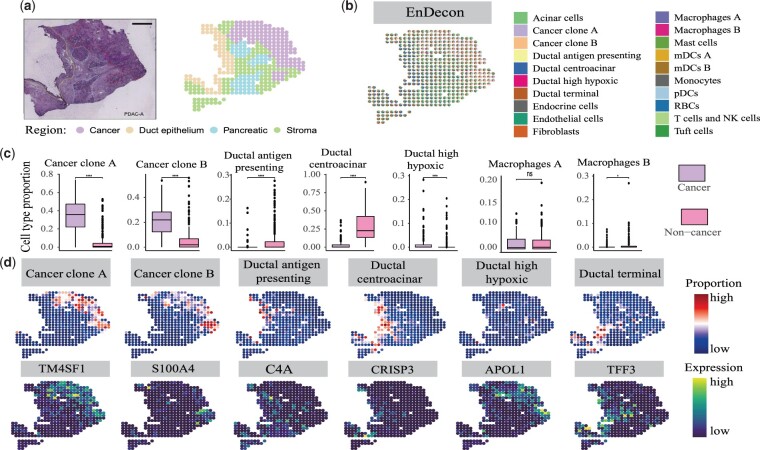
Analysis of the PDAC SRT data. (**a**) Annotation of PDAC tissue slice. H&E staining image of the PDAC (left) displays four regions annotated by histologists from the original study (right). (**b**) Visualization of deconvolution result. A spatial scatter pie chart displays cell-type compositions predicted by EnDecon and each scatter represents a spot in SRT data. (**c**) Comparisons of cell type proportions in cancer and non-cancer regions. The boxplot represents the distribution of cell type proportions in each region. For each cell type, a two-sided Wilcoxon Rank Sum test is used to test the difference. In the diagrams, ‘ns’ represents *P*-value > 0.05, * represents 0.01 < *P*-value ≤ 0.05, and **** represents *P*-value ≤ 1e−4. (**d**) Top, the abundances of cell types by EnDecon are visualized on each spatial location. Bottom, the expression level of the corresponding canonical cell type marker genes is displayed

To validate the performance of EnDecon on real SRT data, we apply it to the human PDAC sample with the sample-matched scRNA-seq dataset (inDrop) as reference data (Fig. 4a). Through deconvolution, EnDecon localizes various pancreatic and tumor cell types to distinct tissue regions (Fig. 4b). We observe that the cell type compositions inferred by EnDecon clearly show overall regional segregation between cancerous and non-cancerous regions, between ductal and stromal regions and between ductal and pancreatic regions. Neoplastic cell types are mainly located in the cancer region, while the normal cell types of the pancreas are mainly located in ductal and pancreatic regions excluded from the tumor regions. In contrast, the compared methods, such as SCDC, SPOTlight and SVR, do not clearly delineate these regional segmentations (Supplementary Fig. S13). The dominant cell types inferred by EnDecon in each spot also show a clear separation between cancerous and non-cancerous regions, whereas most compared methods do not show effectiveness in distinguishing these two regions compared with EnDecon (Supplementary Fig. S14). The two ensemble methods, EnDecon and EnDecon_mean, delineate cancer and non-cancer regions. EnDecon clearly delineates the segmentation between pancreatic interstitial regions, while EnDecon_mean blends these regions together (Supplementary Fig. S14).

To quantify the difference in the proportion of predicted cell types in cancer and non-cancer regions, we first divided the spots into two complementary subsets according to the region in which they are located (cancer and non-cancer regions) and test them using a two-sided Wilcoxon Rank Sum test Differences for each cell type. The distributions of most predicted cell type proportion in these two regions show significant differences (Fig. 4c and Supplementary Fig. S15). The predicted cell type proportion distribution of tumor cells (e.g. cancer clone A and B cells) shows a significant difference between these two regions (Fig. 4c). The proportion of two different macrophage subpopulations predicted by EnDecon reveals different distributions between cancer and non-cancer regions, which may be a key functional feature of cancer tissue compartmentalization (Ma *et al.*, 2022).

Many cell types are localized to specific spatial regions by EnDecon. Examining the agreement between the spatial distribution of specific cell types and expression patterns of matched marker genes confirms the high accuracy predicted by EnDecon (Fig. 4d). In line with the previous results (Moncada *et al.*, 2020), the cancer clone A and B cells are located in two subregions of the cancer region, in which the cancer clone A cells mainly distribute in an upper subregion and the cancer clone B cells mainly distribute in a bottom subregion. These results are consistent with the spatial expression patterns of the marker genes TM4SF1 and S000A4 (Fig. 4d). The proportions of ductal centroacinar cells inferred by EnDecon mainly appear in the ductal epithelium region, consistent with the spatial expression pattern of the marker gene CRISP3 (Röder *et al.*, 2016). Ductal high hypoxic cells are mainly located in the surrounding cancerous part of the tissue, similar to the expression of marker gene APOL1 (Sedlakova *et al.*, 2014). The compositions of terminal ductal cells inferred by EnDecon mainly reside around the ductal epithelium area and pancreatic region, consistent with the expression pattern of the marker gene TFF33 (Rovira *et al.*, 2010).

When examining cell type enrichment/depletion within annotated tissue regions, we observe different regional enrichment patterns of normal pancreatic and tumor cells (Supplementary Fig. S16). The cancer clone A and B cells are enriched in the cancerous region. As expected, all ductal cells are mainly enriched in the duct epithelium region, and only the ductal high hypoxic cells are also enriched in the cancer region (Moncada *et al.*, 2020). We observe that two subpopulations of myeloid dendritic cells, mDCs A and B, appear in different regions. The mDCs A cells are enriched in the pancreatic region while the mDCs B cells are enriched in all regions except for the cancer region (Moncada *et al.*, 2020). These quantified enrichment results are consistent with the cell type localization.

We then depict cell type colocalization map to explore the potential cellular interactions. A well-preserved colocalization pattern of cancer clone A and B cells supports a role in the formation of tissue carcinogenesis (Supplementary Fig. S17). Ductal centroacinar and duct terminal cells are captured with spatial correlation as expected (Means *et al.*, 2001) and show anti-correlation with neoplastic cells. Ductal high hypoxic is positive correlative with neoplastic cells, supporting the role in forming the hypoxic and nutrient-poor tumor microenvironment (Tao *et al.*, 2021). mDCs serve as a bridge linking to immune responses responsible for capturing, processing and presentation of antigens on their surface to T cells, and we observe that mDCs are colocalized with T cells (Chistiakov *et al.*, 2015).

### 4.3 Charting spatial heterogeneity in breast cancer

**Fig. 5.**
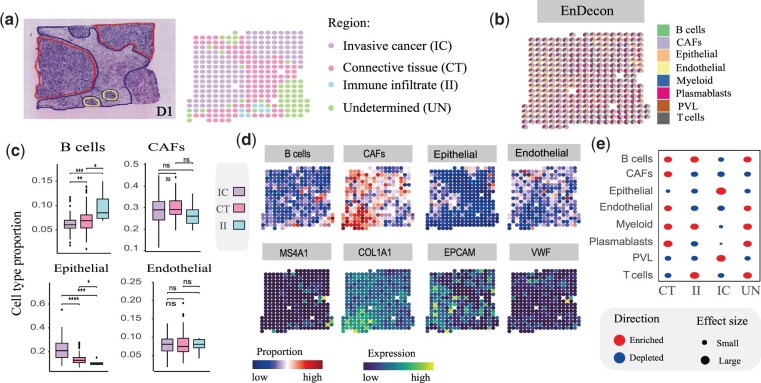
Analysis of the breast cancer SRT data. (**a**) Annotation of breast cancer tissue region. H&E staining image of the breast cancer (left) displays three annotated and an undetermined regions (right), annotated by histologists from the original publication. (**b**) Visualization of deconvolution result. A spatial scatter pie chart displays cell-type compositions predicted by EnDecon and each scatter represents a spot in SRT data. (**c**) Comparisons of cell type proportions in three refined annotated regions. The boxplot represents the distribution of cell type proportions in each region. For each cell type, a two-sided Wilcoxon Rank Sum test is used to test the significance of difference. In the diagrams, ‘ns’ represents *P*-value > 0.05, * represents 0.01 < *P*-value ≤ 0.05, and **** represents *P*-value ≤ 1e−4. (**d**) Top, the spatial distribution of abundances of different cell types estimated by EnDecon. Bottom, the spatial distribution of expression level of the corresponding canonical cell type marker genes. (**e**) Enrichment (red) and depletion (green) of predicted cell types in the four main annotated regions. The enrichment scores are proportional to the size of the circles (A color version of this figure appears in the online version of this article)

To delineate the complex tumor microenvironment, we apply EnDecon to a human breast cancer dataset generated using the ST protocol with published scRNA-seq data from 10× chromium as reference (Fig. 5a). As annotated by a pathologist based on the morphology of the associated H&E staining image, the SRT data consist of three annotated regions [connective tissue (CT), immune infiltrate (II) and invasive cancer (IC) regions] and one undetermined (UN) region. We observe that the cell type compositions of epithelial cells estimated by EnDecon have higher values in the IC region than in other regions (Fig. 5b). The distribution of epithelial cells may form regional segment between IC and CT regions. However, most of the compared methods (e.g. CARD, ‘MuSiC weighted’ and SPOTlight) do not show clearly this regional segregation, which is evidenced by the distribution of cell type proportions within spots (Supplementary Fig. S18). The dominant cell types across spots inferred by EnDecon also clearly delineate regional segmentation between IC and CT regions, which are not captured by most comparison methods, such as CARD, SCDC and SPOTlight (Supplementary Fig. S19).

To further explore the effectiveness of EnDecon in distinguishing cell type compositions across regions, we focus on the cell type compositions of spots located in the well-refined annotated regions [CT (109 spots), IC (139 spots) and II (7 spots)]. We correlate pathologist-annotated regions with predicted cell-type proportions to quantify differences in the distribution of cell-type proportions across tissue regions. By dividing the spots into three subsets based on their regions, we observe that the spatial distributions of the most inferred cell type proportions show significant differences in different regions (Fig. 5c and Supplementary Fig. S20). The predicted spatial distributions of immune B and T cells show significant differences between the II and the other regions. In contrast, the proportion of epithelial cells was greatest in the IC area and the smallest in the IC area. The proportion of CAFs cells varied slightly between regions, being minimal in region II. Endothelial cells are evenly distributed throughout the tissue.

To further validate the accuracy of the predicted cell type compositions by EnDecon, we compare the spatial distribution of cell types with that of the corresponding marker gene expression levels (Fig. 5d and Supplementary Fig. S21). The results confirm the high accuracy of the predicted cell type compositions. For example, B cells are mainly distributed in II and UN regions, and its marker gene, MS4A1, highly expressed in the two regions. A marker gene of CAFs, COL1A1, shows clearly expression pattern in the CT region, consistent with the distribution of CAF cells. The located region of epithelial cells is consistent with the expression pattern of the corresponding marker gene EPCAM.

We examine the accuracy of predicted cell type spatial mapping by performing region-based cell type enrichment analysis. Cell types are enriched in diverse regions, which are matched with the cell type abundances inferred by EnDecon (Fig. 5e). Epithelial and PVL cells are enriched in the IC region, and CAFs are enriched in the CT region. These enrichment results further illustrate the accuracy of EnDecon in predicted cell-type compositions.

The cell type colocalization map is also characterized (Supplementary Fig. S22). Two cell types are regarded as colocalization in a spatial context when the corresponding spot-wise PCC score is positive. In the immune cell populations, B cells are anti-correlated with plasmablasts cells, reflecting that the B cells and plasmablasts cells reside in distinct regions within tumor (Öhlund *et al.*, 2017). In contrast, B cells and T cells exhibit strong colocalization signal and both are enriched in the II region (Fig. 5d and e). On the other hand, T cells are colocalized with myeloid cells (Supplementary Fig. S22), which agrees with the fact that they often interact with each other in the tumor microenvironment (DeNardo *et al.*, 2010). Epithelial and PVL cells are colocalized with each other, and both anticorrelate with the other cell types as expected.

## 5 Discussion

By integrating the base deconvolution results produced by 14 individual cell-type deconvolution methods, we propose a new weighted ensemble learning method to predict cell-type compositions for SRT data. The superior performance of our method on simulated datasets and a real adult mouse brain dataset demonstrates the accuracy of EnDecon in cell-type deconvolution. We also apply our method to three real SRT data to explore the spatial distributions of cell types on those tissues. Mapping cell type compositions predicted by EnDecon into spatial context, our method can successfully segment tissue regions predefined by pathologists. EnDecon correctly locates cell type to the specific spatial regions, which are consistent with the gene expression patterns of the corresponding cell type marker genes. Furthermore, cell type-enriched regions are in line with those of located regions. These comprehensive analysis results confirm the effectiveness of EnDecon in predicting cell type compositions within spots for SRT data.

We find that individual methods such as Cell2location, DWLS, ‘MuSiC weighted’ and RCTD tend to have better performance on different datasets (Supplementary Fig. S25), which is partly consistent with previous benchmark studies (Chen *et al.*, 2022; Li *et al.*, 2022a). However, none of them is an apparent winner across all datasets. EnDecon outperforms all individual methods in almost all cases, suggesting that using ensemble learning to integrate different deconvolution methods is more reasonable than selecting better-performing methods. In addition, it would be a good idea to remove poorly performing methods before integrating them. We do not take this step for the following reasons. First, few methods perform poorly in all situations, and a method that performs poorly in some situations may perform well in others. Second, in real data applications, we often do not have the ground truth of the proportion of cell types within each spot, so we cannot directly quantify the performance of individual methods. Third, our weighted approach is developed to handle situations where the performance of different individual methods varies widely for a considered dataset. Our method can automatically compute weights for each integrated method and combine the base deconvolution results based on the weights. Experiment results show that the weights assigned by EnDecon to base deconvolution methods have a significant positive correlation with their performance, indicating that EnDecon can automatically increase the weights of better-performing methods and decrease the weights of poorer-performing methods without ground truth.

To show the effectiveness of our weighted ensemble approach, we have compared EnDecon with a baseline ensemble approach, EnDecon_mean, which treats each base deconvolution result equally and uses their average as the ensemble results. Although the performance difference between EnDecon and EnDecon_mean is relatively small in some cases, our weighted approach would be more reasonable. First, EnDecon outperforms EnDecon_mean in 16 out of 18 simulation results (6 datasets × 3 metrics) (Supplementary Fig. S25). Second, EnDecon can provide more meaningful and reasonable results than EnDecon_mean in real data experiments. For example, EnDecon can correctly capture glial cells outside the mouse brain that are incorrectly predicted as neuron cells by EnDecon_mean, and the neuron cells in the hippocampal region that are incorrectly predicted as glial cells by EnDecon_mean (Fig. 3b). In addition, the dominant cell types within spots inferred by EnDecon can clearly delineate the segmentation between pancreatic interstitial regions, while the dominant cell types inferred by EnDecon_mean blend these regions together (Supplementary Fig. S14). Third, compared to EnDecon_mean, EnDecon can compute a weight for each base method. Experiment results have shown that the weights assigned to base methods are positively correlated with their performance on both simulated and real datasets. Therefore, the weights that EnDecon assigns to the base deconvolution methods are meaningful, as they may provide clues about the performance of the base deconvolution methods when the ground truth is unavailable.

It is important to note that the performance of an individual method may vary across different settings. Running with different settings of the individual method may improve its and EnDecon’s performance. However, we adopt default settings suggested by the authors for each base deconvolution method and do not explore different settings for the following reasons. First, the default settings suggested by the authors may have been tested extensively by them, which may generalize well to new test scenarios. Second, each individual method may have multiple parameters that need to be set, making it impractical to try all possible combinations of parameter settings for the 14 individual methods. Third, a good method should not be very sensitive to parameter settings, and most previous benchmark studies (Chen *et al.*, 2022; Li *et al.*, 2022a) and research articles also adopt default settings. Fourth, when ground truth is available, we can choose the best settings in terms of accuracy. However, in most real data applications, we usually do not have a metric score to choose the optimal settings since this is no ground truth of cell type compositions. Fifth, our goal is to develop an easy-to-use, robust and accurate ensemble method that can generalize to new datasets without fine-tuning the base method settings. Our EnDecon will be inconvenient to use if we let the users try different settings of the base methods.

EnDecon will be improved in two directions in the future. First, while we have endeavored to include more of the currently available individual deconvolution methods in EnDecon, cell-type deconvolution of SRT data is a rapidly developing field that will soon yield more effective and efficient methods. Integrating more individual deconvolution methods into EnDecon will not only improve the accuracy of deconvolution but also make it more robust to noise in SRT data and batch effects between SRT data and reference scRNA-seq datasets. Thus, we will integrate more individual methods into EnDecon. Second, the rapid development of scRNA-seq technologies allows us to have multiple reference scRNA-seq datasets from different platforms or samples for the same tissue. Borrowing strength from multiple reference datasets may increase the accuracy and robustness. Currently, EnDecon and all individual methods it includes are based on only a single reference scRNA-seq dataset. In the future, we will extend our EnDecon to better integrate the complementary strengths between different scRNA-seq reference datasets.

## Supplementary Material

btac825_Supplementary_Data

## Data Availability

The datasets are derived from sources in the public domain: the adult mouse brain and mouse brain cortex SRT data are obtained from the 10x Genomics websites (https://www.10xgenomics.com/resources/datasets/adult-mouse-brain-section-2-coronal-stains-dapi-anti-gfap-anti-neu-n-1-standard-1-1-0 and https://www.10xgenomics.com/resources/datasets/mouse-brain-section-coronal-1-standard-1-1-0) and the corresponding scRNA-seq data from the Gene Expression Omnibus (GEO) website under accession number ID GSE71585, the human pancreatic ductal adenocarcinoma (PDAC) SRT and the corresponding scRNA-seq data from the GEO website under accession number ID GSE111672, the human breast cancer SRT data from the Zenodo data repository (https://doi.org/10.5281/zenodo.4739739) and the corresponding scRNA-seq data from the GEO website under accession number ID GSM5354515.
