# Supplementary material for: EnDecon: cell type deconvolution of spatially resolved transcriptomics data via ensemble learning
Source: Bioinformatics. 2022 Dec 22;39(1):btac825. doi: 10.1093/bioinformatics/btac825 (PMC9825263; doi:10.1093/bioinformatics/btac825)
Supplement: btac825_Supplementary_Data [file btac825_supplementary_data.pdf]

EnDecon: cell type deconvolution of spatially resolved transcriptomics data via ensemble learning

Jia-Juan Tu, Hui-Sheng Li, Hong Yan and Xiao-Fei Zhang

Contents

1 Supplementary Figures **2**

2 Supplementary Tables **24**

3 Supplementary Text **28**
3.1 Implementation of the individual deconvolution methods 28
3.1.1 CARD 28
3.1.2 Cell2location 28
3.1.3 DeconRNASeq 28
3.1.4 DestVI 28
3.1.5 DWLS and SVR 29
3.1.6 MuSiC 29
3.1.7 RCTD 29
3.1.8 SCDC 30
3.1.9 SpatialDWLS 30
3.1.10 SPOTlight 30
3.1.11 Stereoscope 30
3.1.12 STdeconvolve 31
3.2 Summary of EnDecon 31
3.3 Simulation data analysis 32
3.3.1 Generation of simulated SRT data 32
3.3.2 Effect of sample sizes of the reference scRNA-seq data on the performance of deconvolution 33
3.3.3 Running time comparison 34
3.4 Real SRT data analysis 35
3.4.1 Collecting real SRT data and reference scRNA-seq dataset 35
3.4.2 Performance evaluation on real SRT data 37
3.4.3 Mouse brain cortex deconvolution 37

1 Supplementary Figures

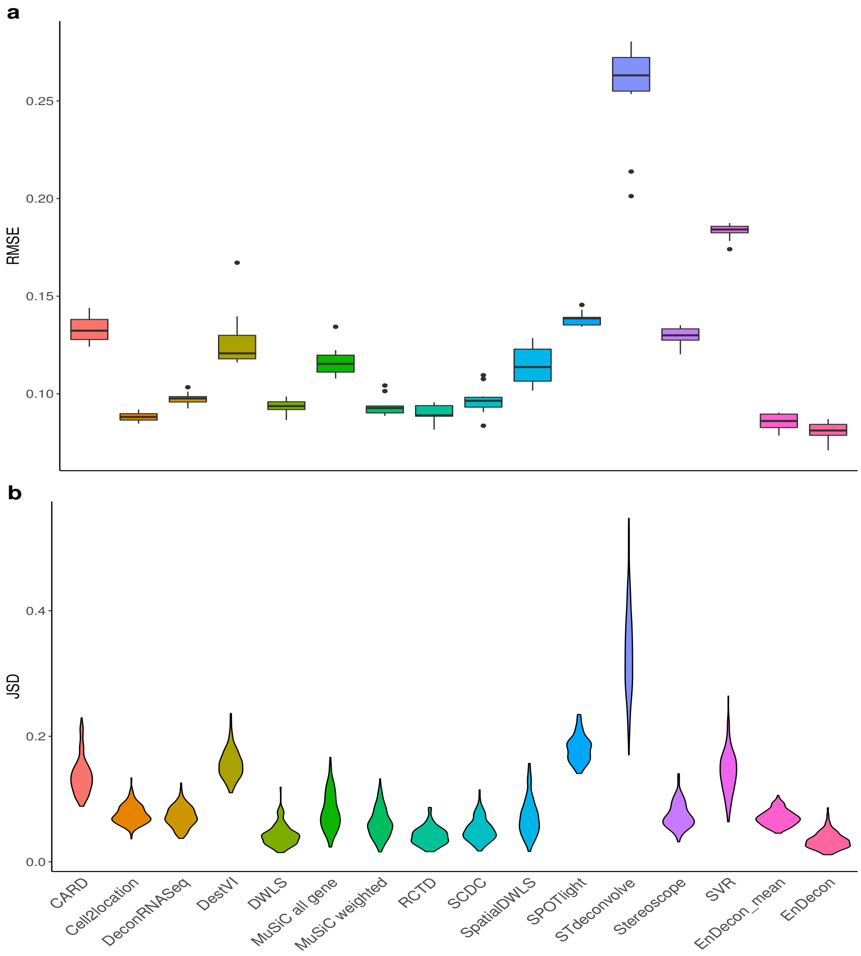

Figure S1: Analysis of the simulation SRT data generated from pancreas scRNA-seq data in scenario 1. The subfigure corresponds to the performance of the deconvolution methods on single evaluation metric. In subfigures, each color represents a deconvolution method. The results are averaged over 10 random generations of the data.

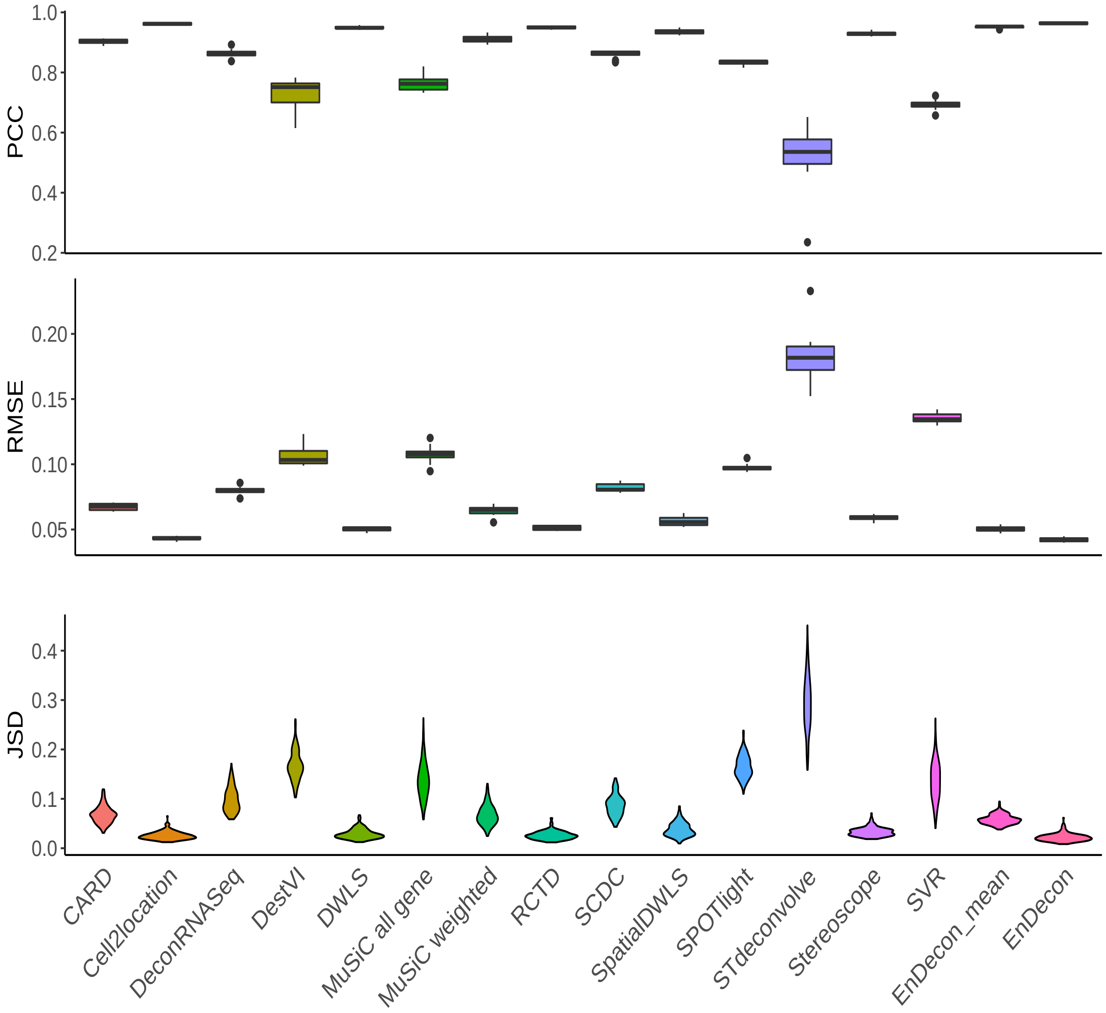

Figure S2: Analysis of the simulation SRT data generated from ovarian cancer scRNA-seq data in scenario 1. The subfigure corresponds to the performance of the deconvolution methods on single evaluation metric. In subfigures, each color represents a deconvolution method. The results are averaged over 10 random generations of the data.

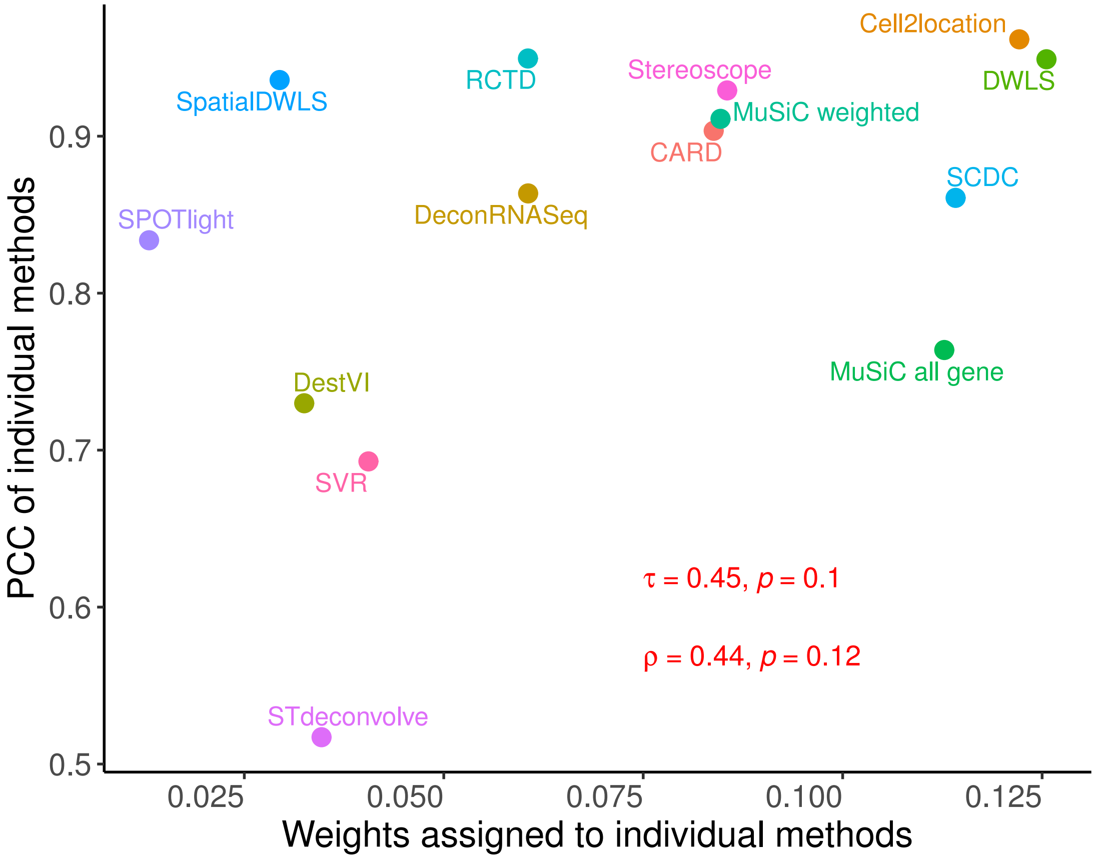

Figure S3: Correlation analysis between the weights (x-axis) inferred by EnDecon and the PCC scores (y-axis) of the base deconvolution methods on ovarian cancer data in scenario 1. The dot presents a base deconvolution method. The Pearson correlation coefficient (τ) and Spearman correlation coefficient (ρ) between the learned weights and PCC scores of base methods, and the corresponding p values (from one-sided t-test) are provided.

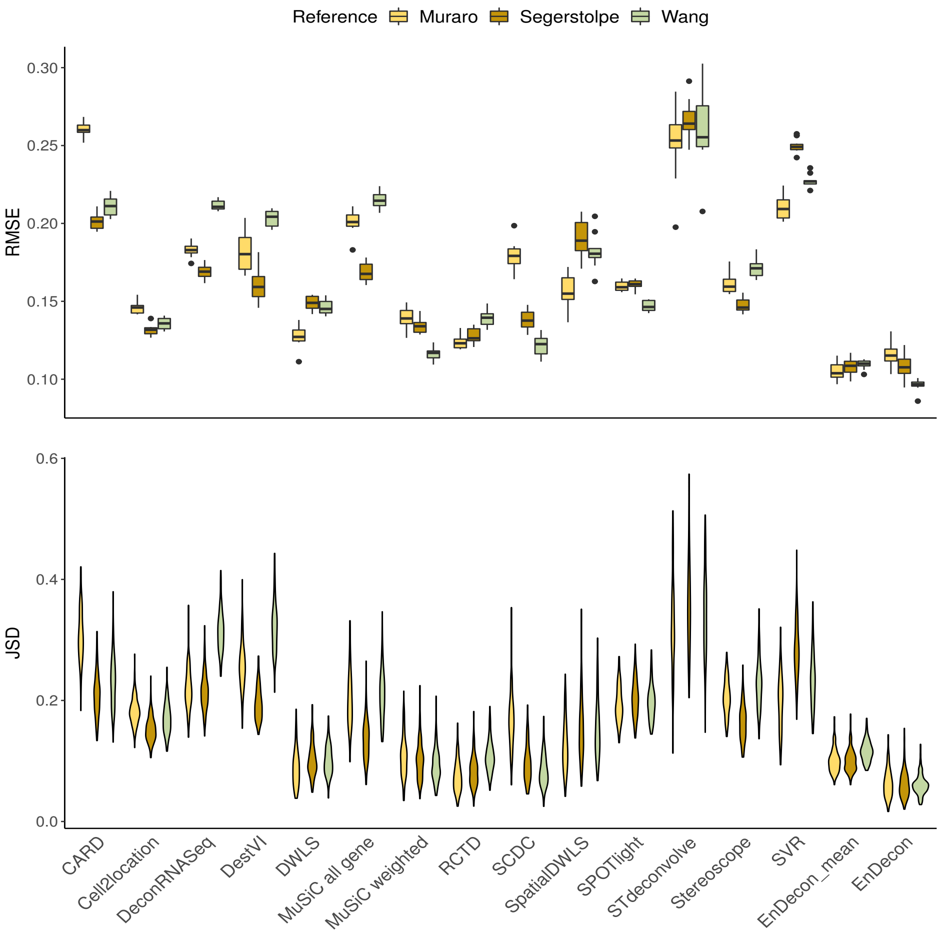

Figure S4: Performance of the deconvolution methods on simulated SRT data with reference datasets from different techniques in scenario 2. The subfigure corresponds to the performace of the methods on single evaluation metric. In subfigures, each color represents different reference scRNA-seq datasets generated from different techniques. The results are averaged over 10 random generations of the data.

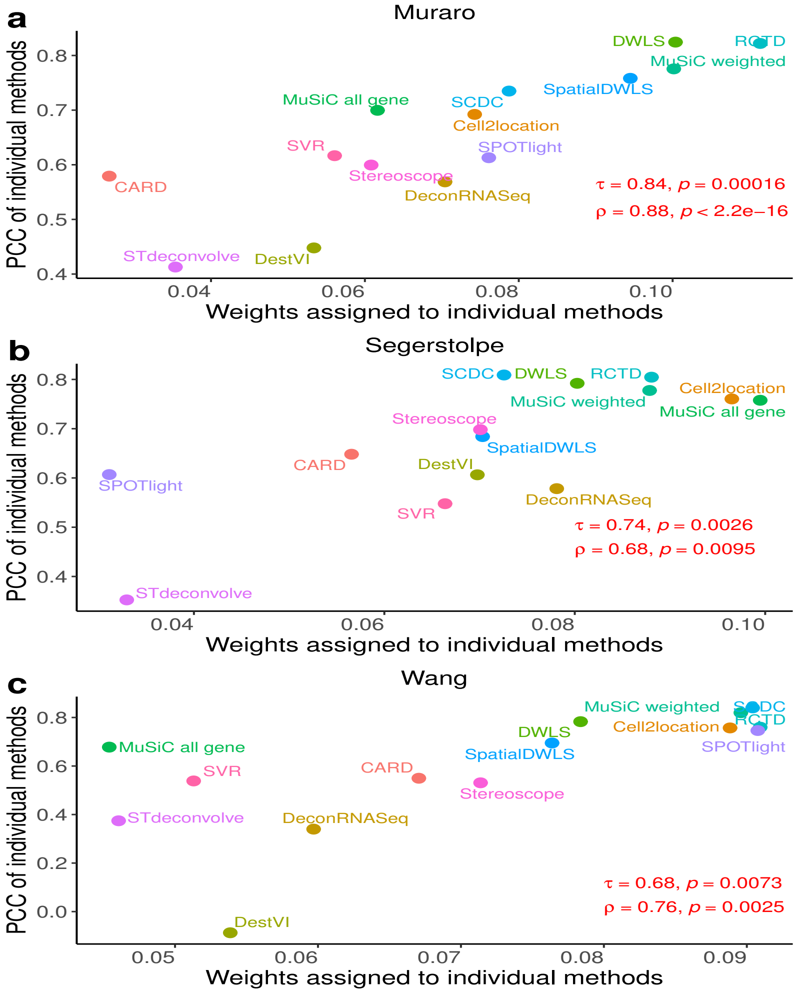

Figure S5: Correlation analysis between the weights (x-axis) inferred by EnDecon and the PCC scores (y-axis) of the base deconvolution methods in scenario 2. The subfigure corresponds to the reference scRNA-seq dataset generated from different techniques. In subfigures, each dot presents a base deconvolution method. The Pearson correlation coefficient (τ) and Spearman correlation coefficient (ρ) between the weights and PCC scores, and the corresponding p values (from one-sided t-test) are provided.

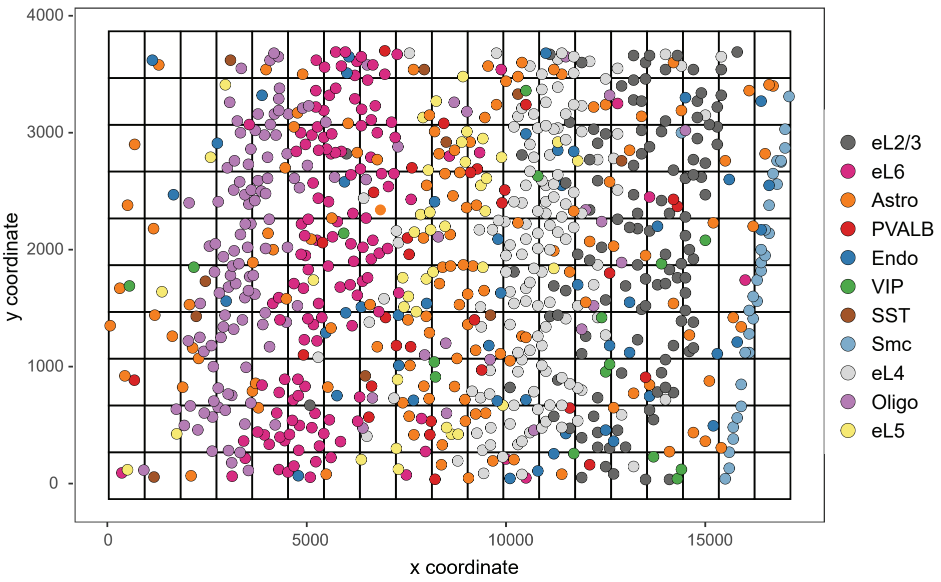

Figure S6: Visualization of cell type spatial distribution for STARmap mouse visual cortex in scenario 3. Each grid represents a simulated spot consisting of multiple cells. Each color represents a cell type. There are 11 cell types, containing 4 excitatory neurons (eL2/3, eL4, eL5, and eL6), Astro (astrocytes), Endo (endothelial), Micro (microglia), Oligo (oligodendrocytes) and Smc (smooth muscle cells).

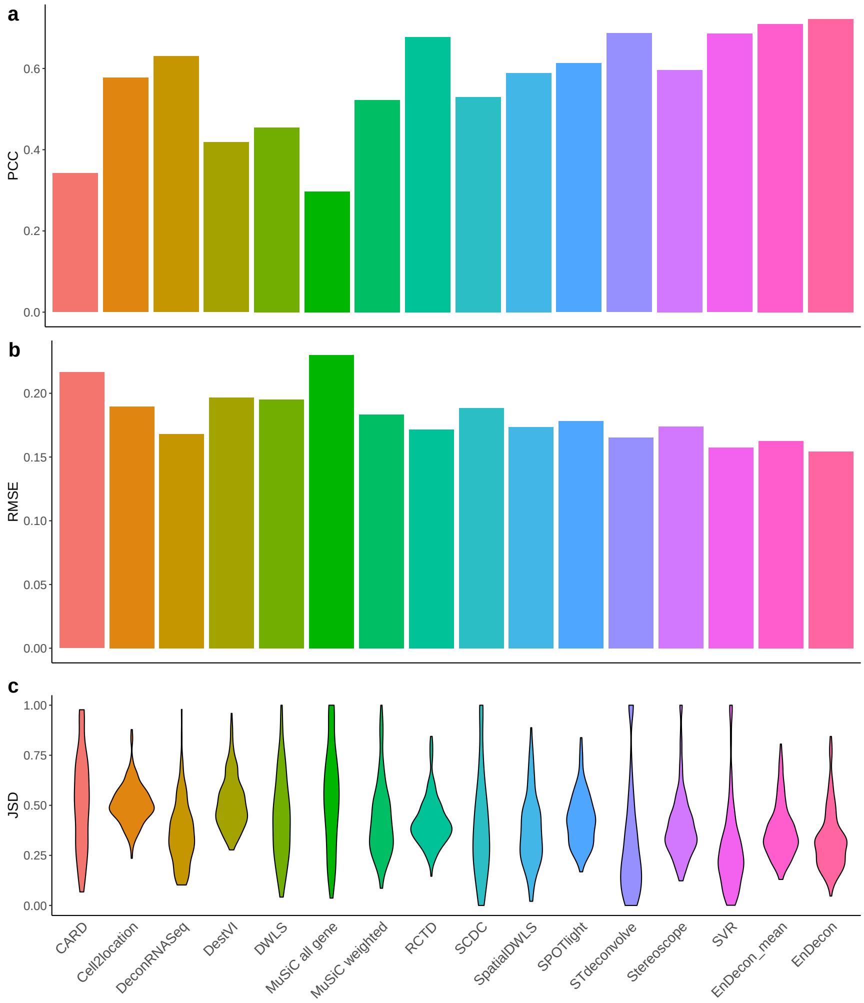

Figure S7: Analysis of the simulation SRT data in scenario 3. The subfigure corresponds to the performance of the deconvolution methods on simulation data with single evaluation metric. In subfigures, each color represents a deconvolution method.

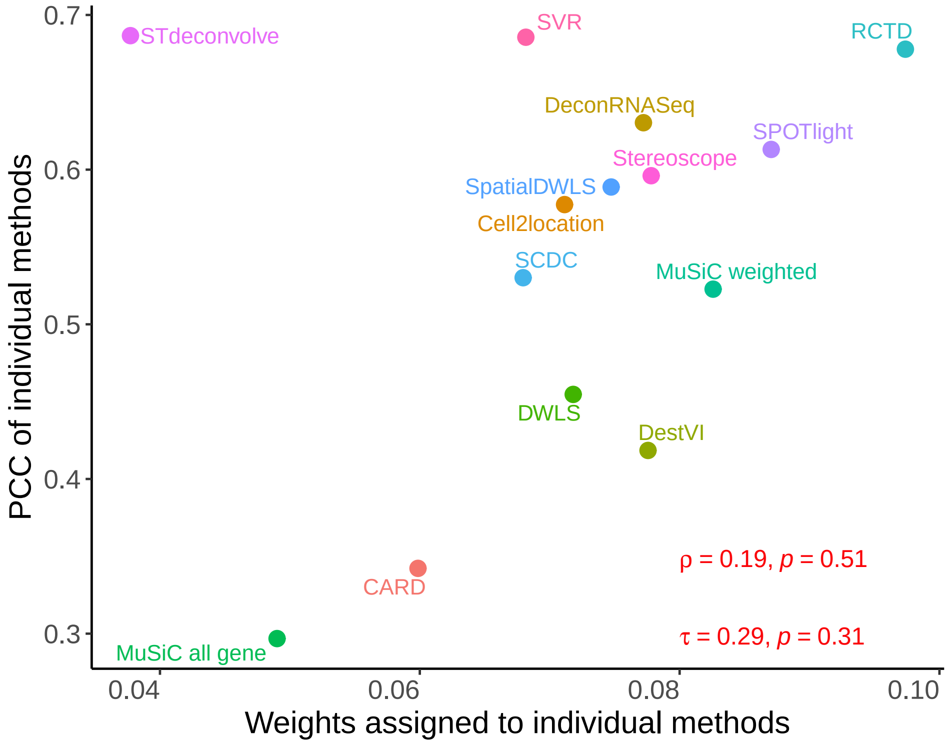

Figure S8: Correlation analysis between the weights (x-axis) inferred by EnDecon and the PCC scores (y-axis) of the base deconvolution methods in scenario 3. The dot presents a deconvolution method. The Pearson correlation coefficient (τ) and Spearman correlation coefficient (ρ) between the weights and PCC scores, and the corresponding p values (from one-sided t-test) are provided.

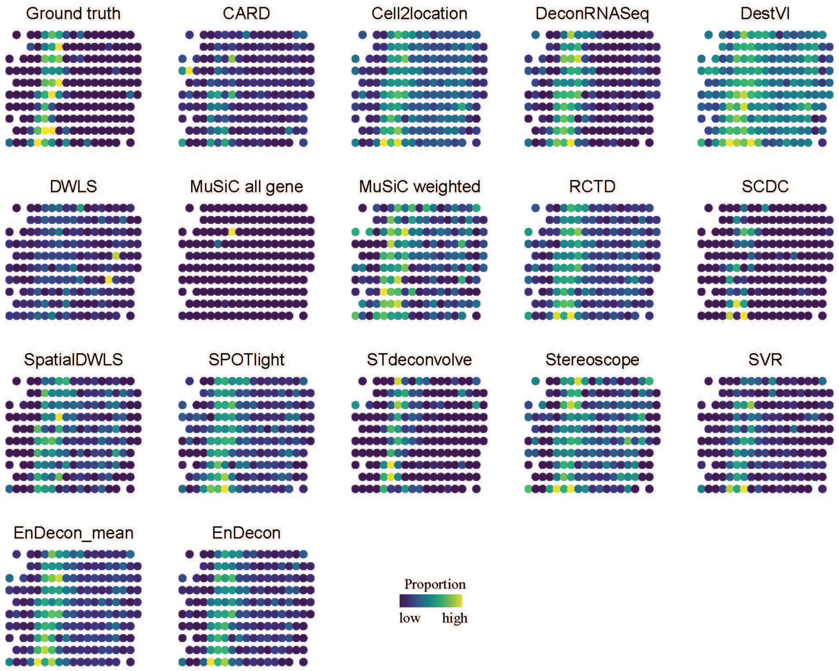

Figure S9: Visualization of the proportions of L4 excitatory neurons within spots simulated from STARmap data, including the ground truth, predicted deconvolution results from base deconvolution methods, EnDecon_mean and EnDecon in scenario 3.

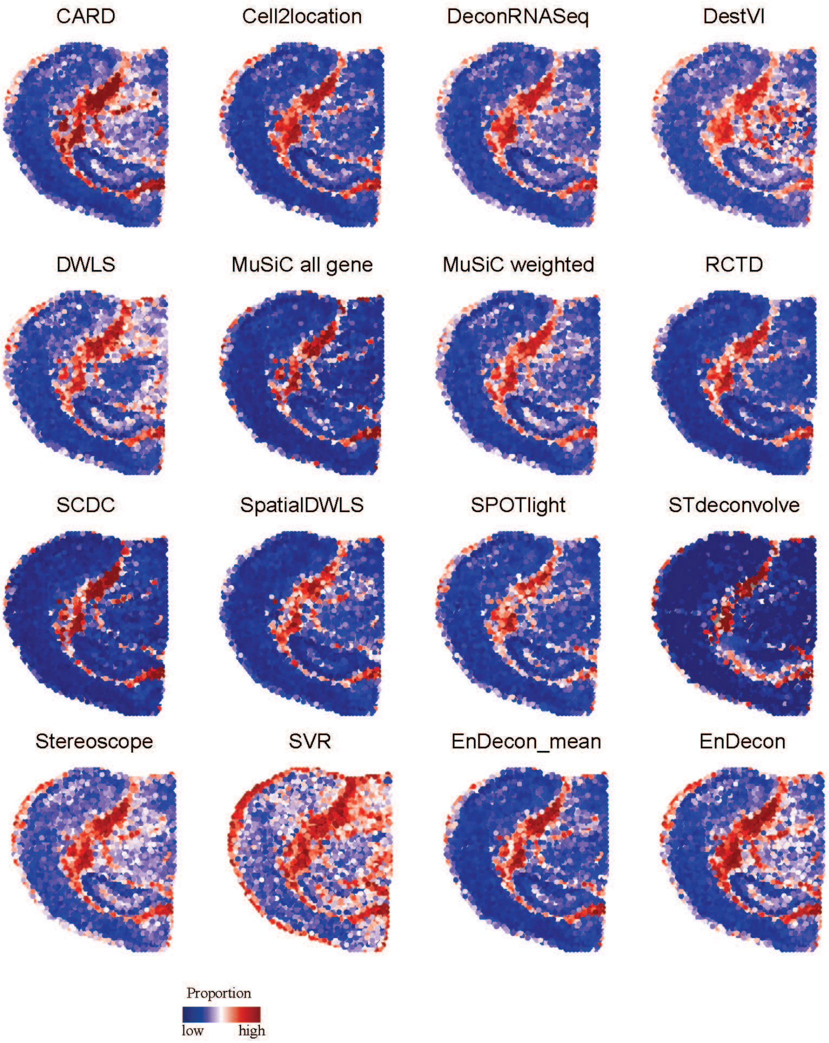

Figure S10: Visualization of the deconvolution results inferred by the deconvolution methods from adult mouse brain SRT data of glial cells. The spatial scatter pie chart displays cell type compositions predicted by the deconvolution method and the scatter represents a spot in SRT data.

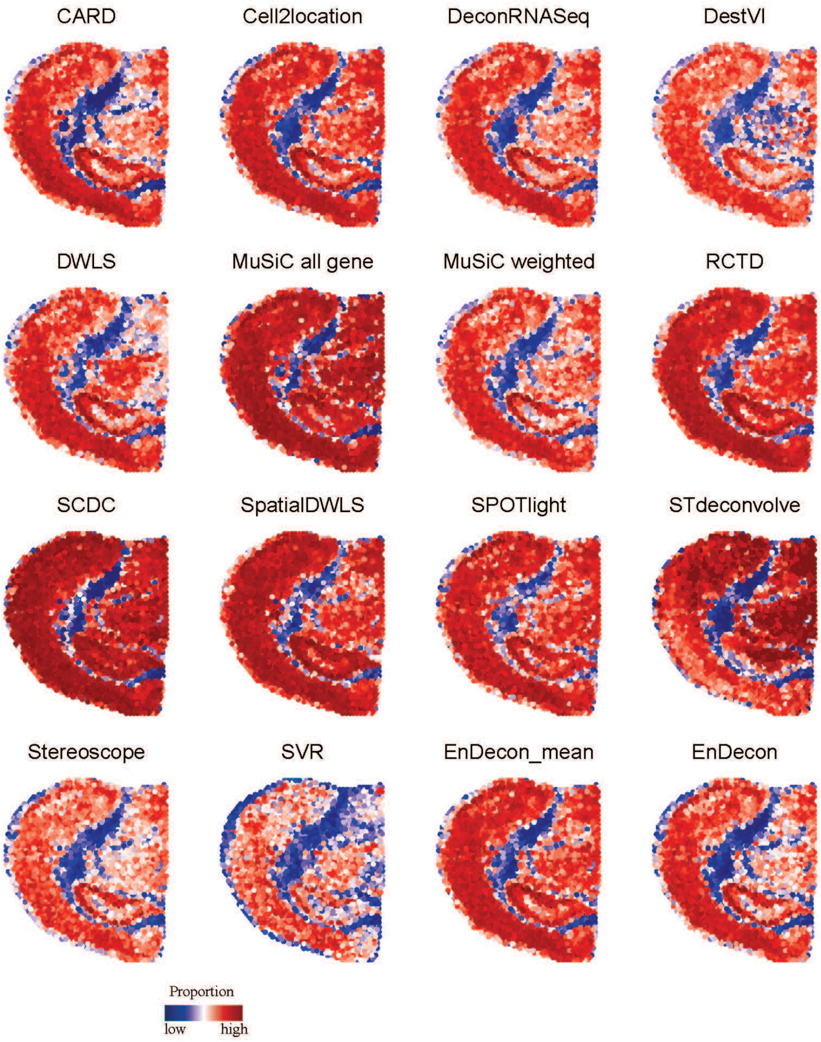

Figure S11: Visualization of the deconvolution results inferred by the deconvolution methods from adult mouse brain SRT data of neuron cells. The spatial scatter pie chart displays cell type compositions predicted by the deconvolution method and the scatter represents a spot in SRT data.

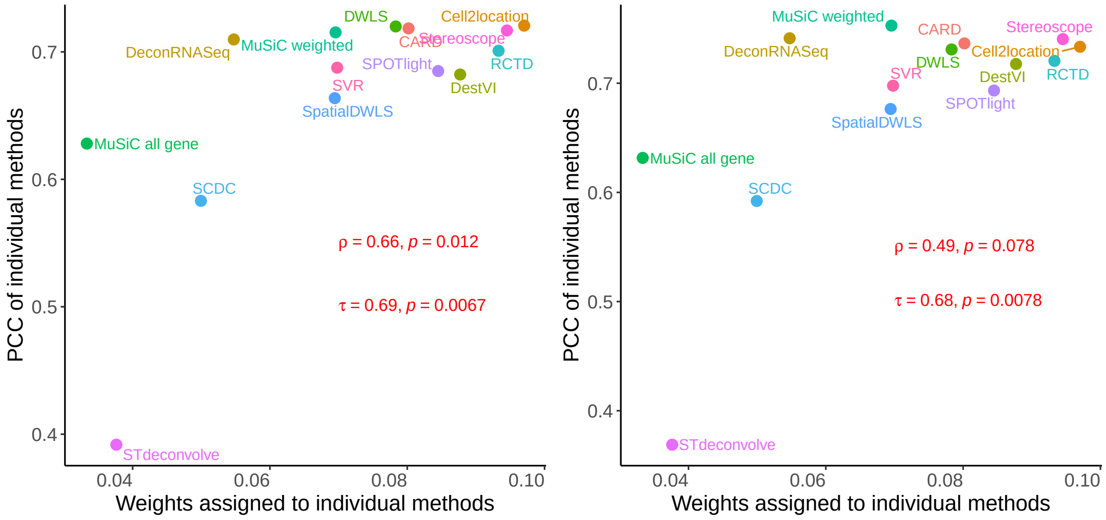

Figure S12: Correlation analysis between the weights (x-axis) inferred by EnDecon and the PCC scores (y-axis) of the base deconvolution methods from adult mouse brain SRT data. The dot presents an individual deconvolution method. The Pearson correlation coefficient (τ) and Spearman correlation coefficient (ρ) between the weights and PCC scores, and the corresponding p values (from one-sided t-test) are provided.

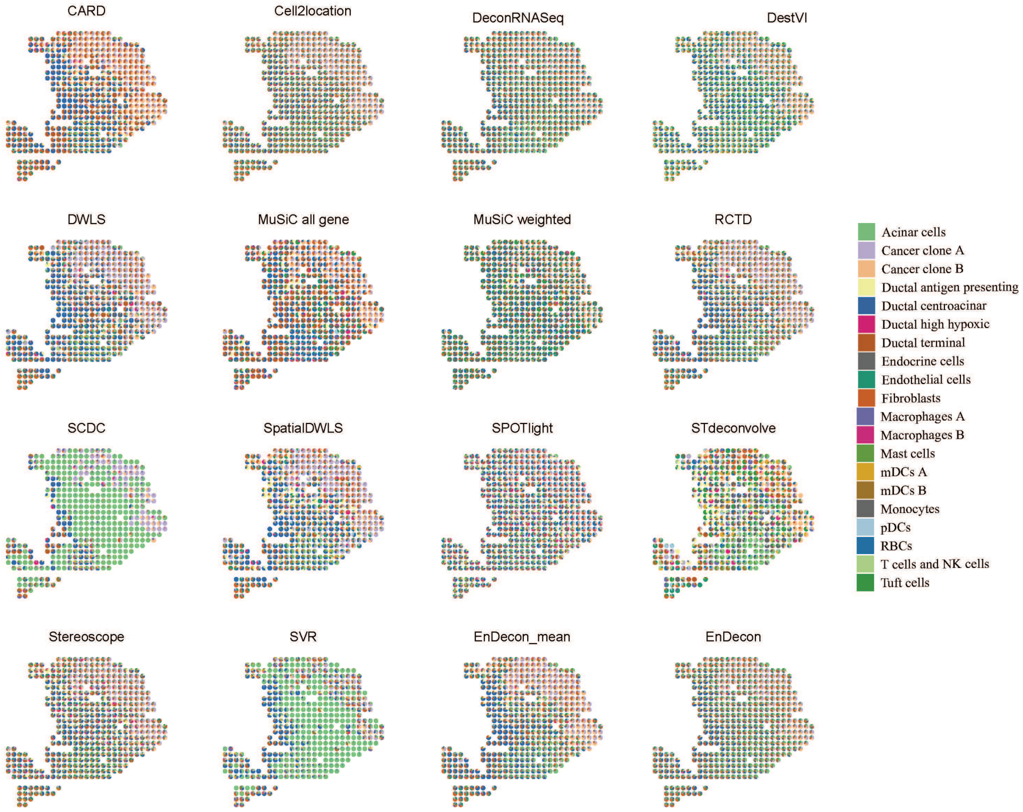

Figure S13: Visualization of deconvolution results inferred by the deconvolution methods from PDAC SRT data. The spatial scatter pie chart displays cell type compositions predicted by the deconvolution method and the scatter represents a spot in SRT data.

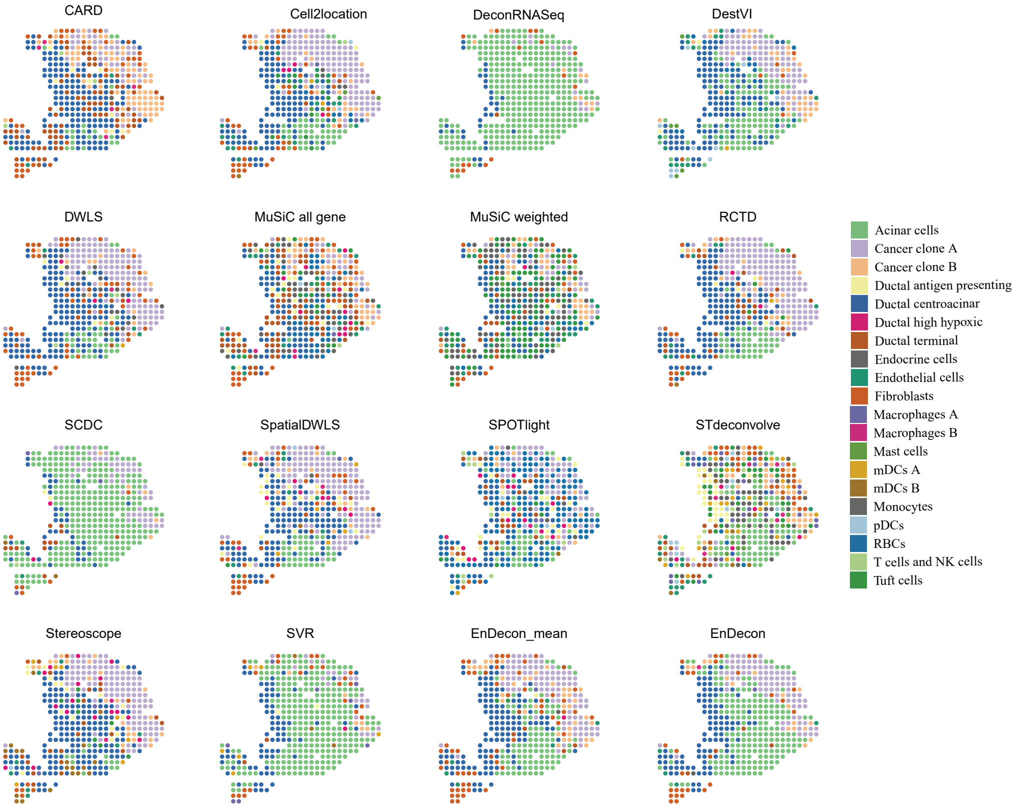

Figure S14: Visualization of dominant cell types inferred by the deconvolution methods from PDAC SRT data. The spatial scatter pie chart displays the spatial distribution of dominant cell types on spot by the deconvolution method.

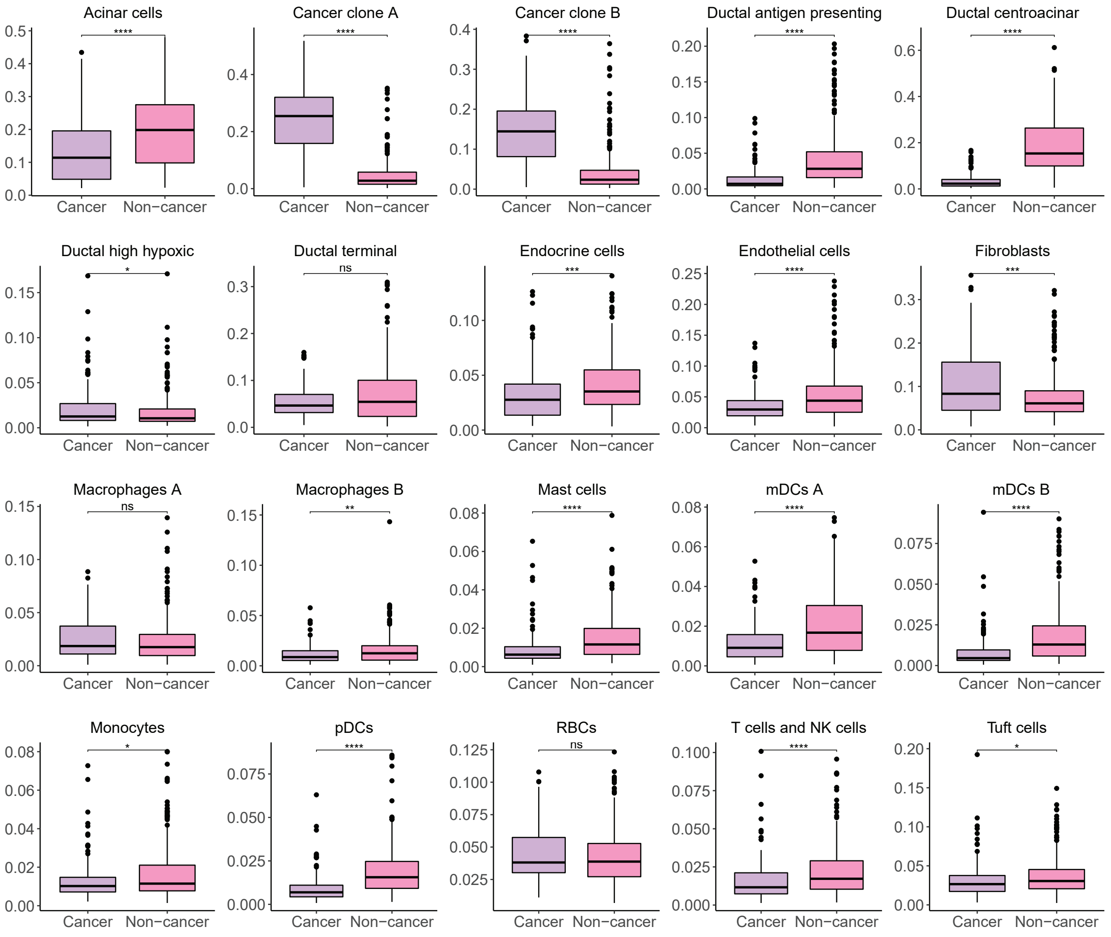

Figure S15: Comparisons of cell type proportions in cancer and non-cancer regions from PDAC SRT data. The boxplot represents the distribution of cell type proportions in each region. Boxes mark the median by a horizontal black line. In the diagrams, "ns" represents p value > 0.05, $\star$ represents $0.01 < p$ value ≤ 0.05, and $\star\star\star\star$ represents $p$value $\leq$ 1e-4.

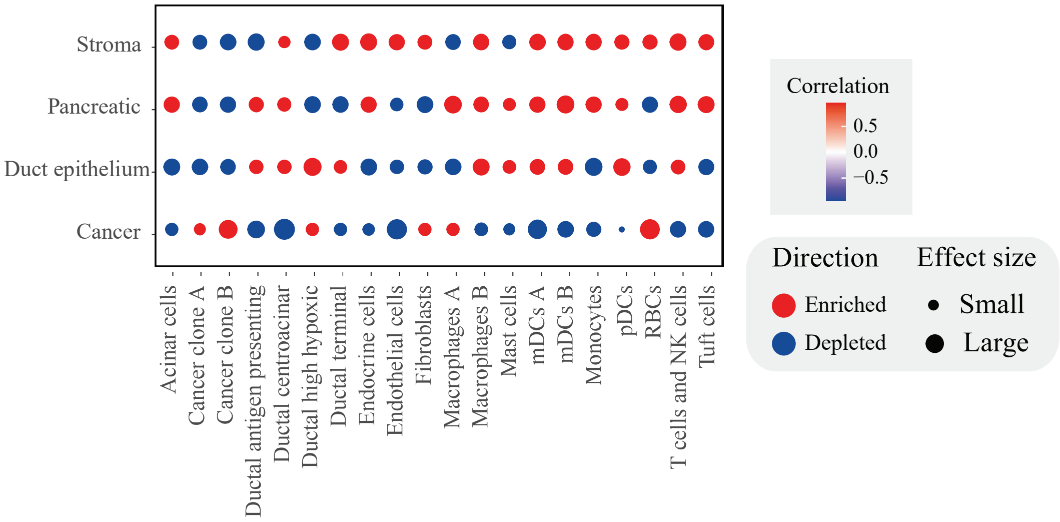

Figure S16: Enrichment (red) and depletion (green) of predicted cell types in the four main annotated regions from PDAC SRT data. The enrichment scores are proportional to the size of the circles (referred as effect size).

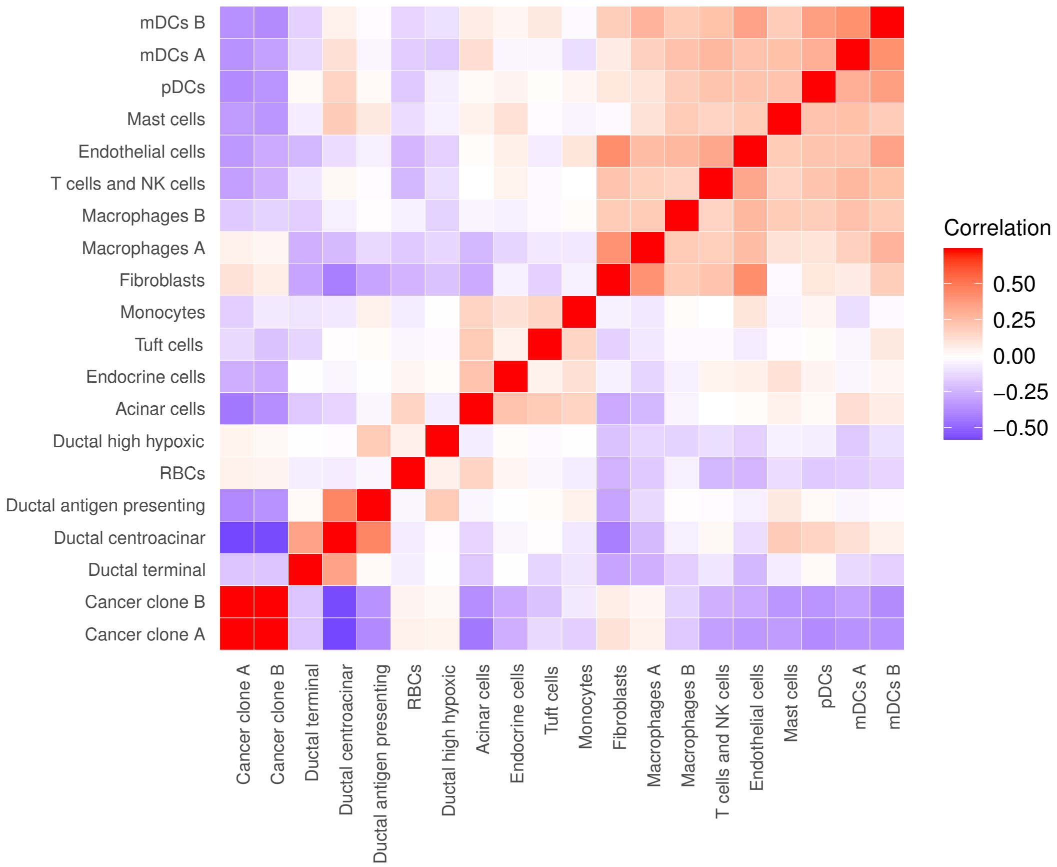

Figure S17: Spatial colocalization map of predicted cell type by EnDecon from PDAC SRT data. Correlation plot shows a different correlation across diverse cell types.

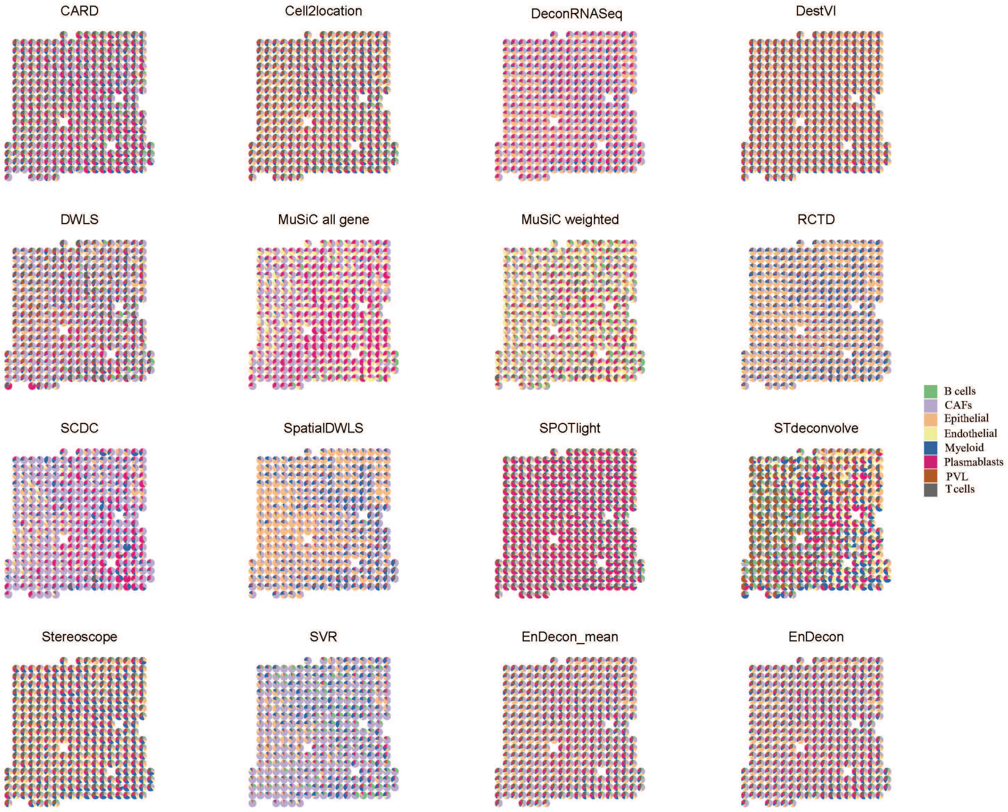

Figure S18: Visualization of deconvolution results inferred by the deconvolution methods from breast cancer SRT data. The spatial scatter pie chart displays cell type compositions predicted by the deconvolution method and the scatter represents a spot in SRT data.

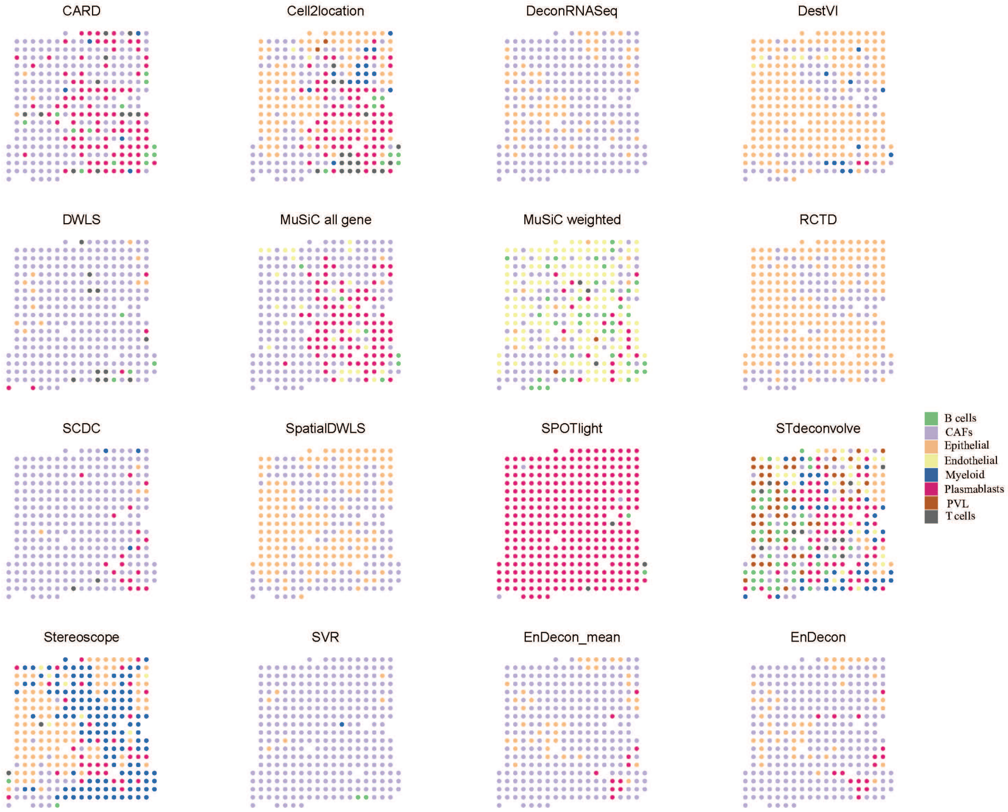

Figure S19: Visualization of dominant cell types inferred by the deconvolution methods from breast cancer SRT data. The spatial scatter chart displays the spatial distribution of dominant cell types on spot by the deconvolution method.

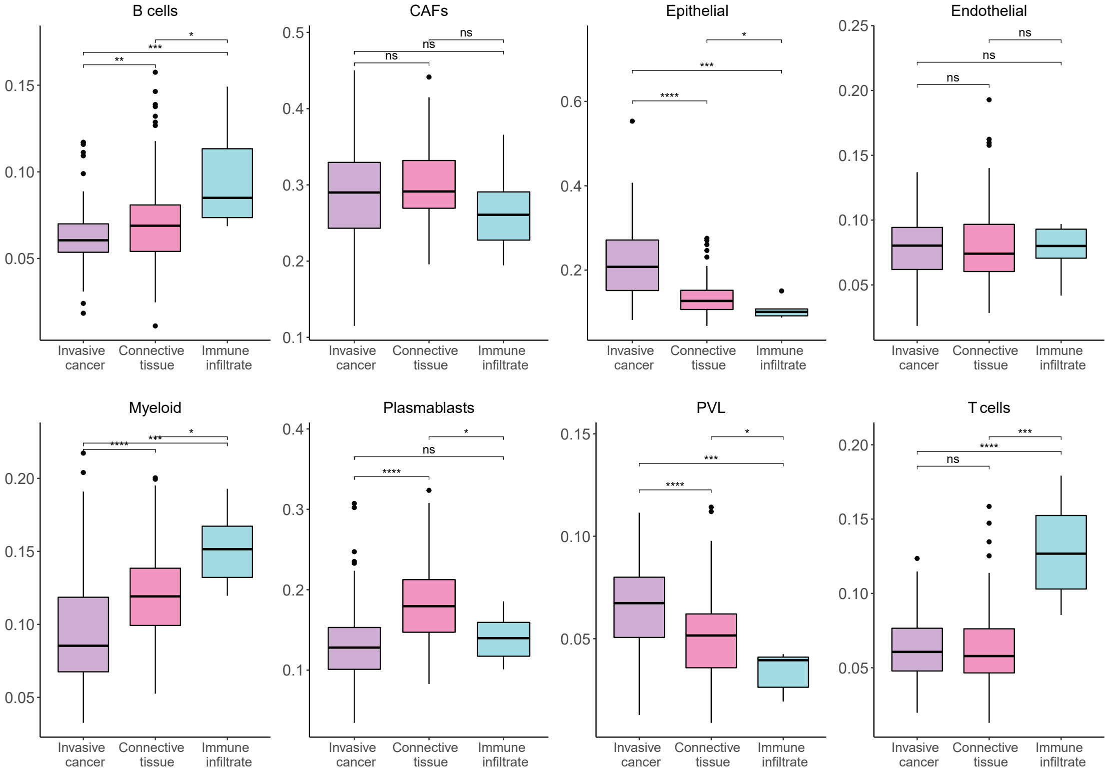

Figure S20: Comparisons of cell type proportions in three refined annotated regions in breast cancer SRT data. The boxplot represents the distribution of cell type proportions in each region. Boxes mark the median by a horizontal black line. In the diagrams, "ns" represents p value > 0.05, $\star$ represents $0.01 < p$ value ≤ 0.05, and $\star\star\star\star$ represents $p$value $\leq$ 1e-4.

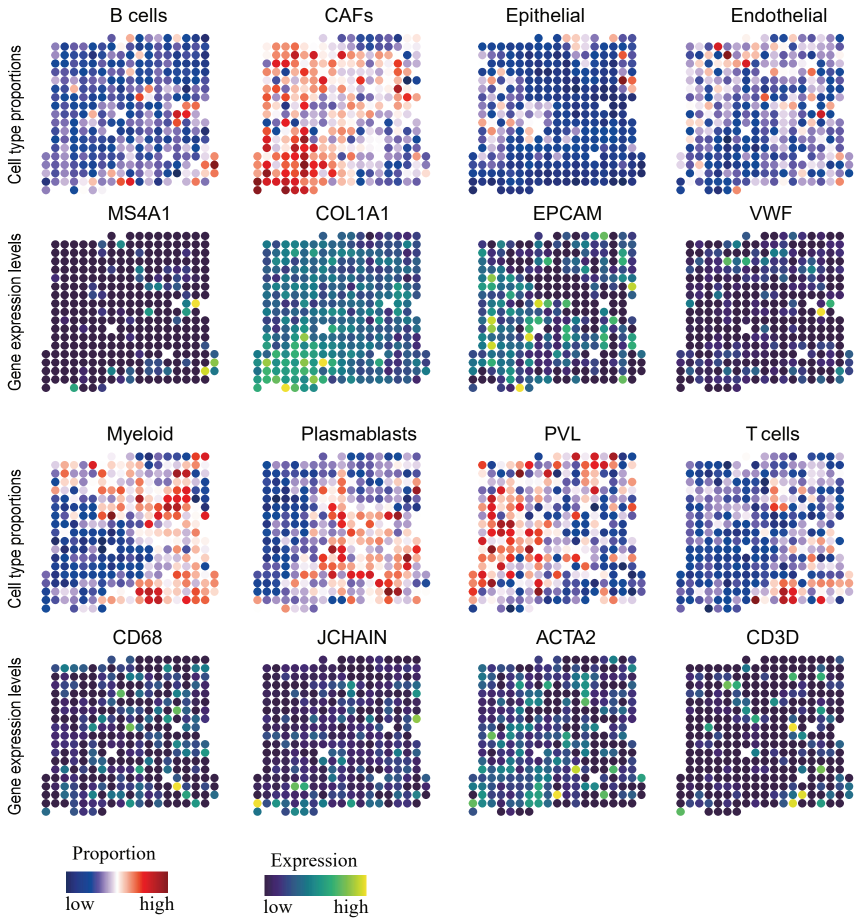

Figure S21: Visualization of the proportion of each cell type estimated by EnDecon and the corresponding canonical cell type marker genes on each spatial location from breast cancer SRT data.

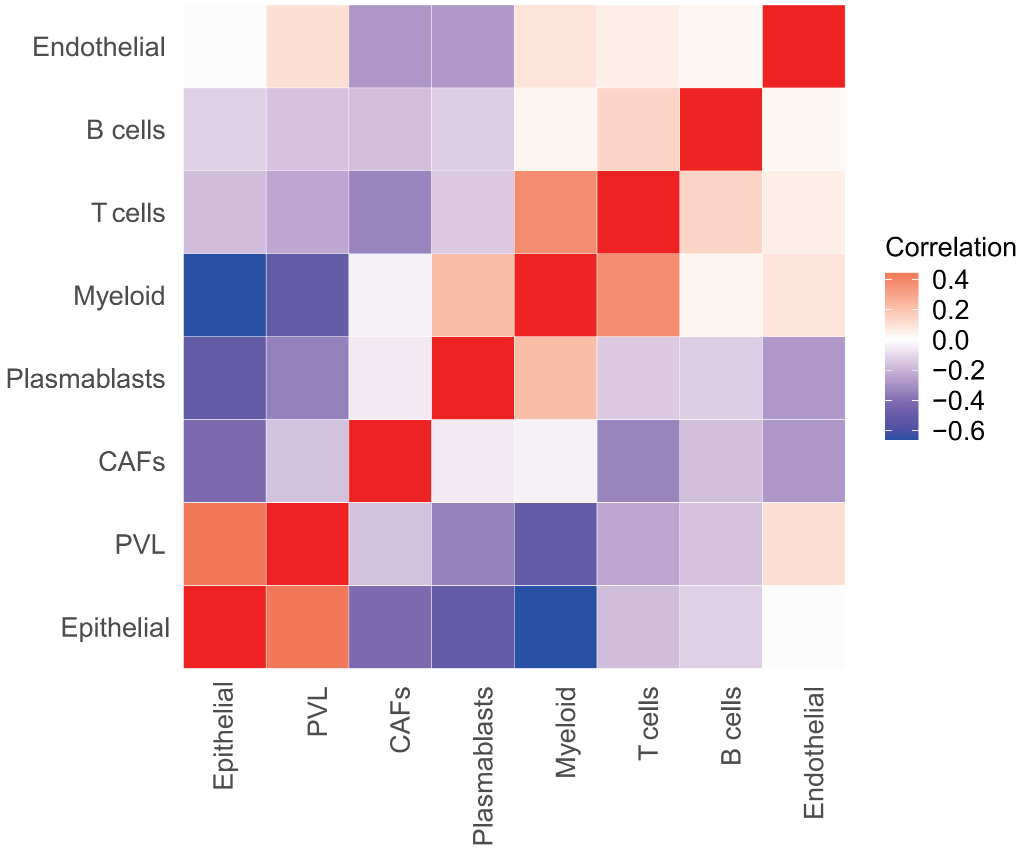

Figure S22: Spatial colocalization map of predicted cell type by EnDecon from breast cancer SRT data. Correlation plot shows a different correlation across diverse cell types.

2 Supplementary Tables

Table S1: Summary of cell type deconvolution methods in EnDecon

Methods	Models	Designed for spatial RNA-seq dataset	Website	Language
CARD [1]	Regression-based model; non-negative matrix factorization model	✓	https://github.com/YingMa0107/CARD	R
Cell2location [2]	Probability distribution-based model; negative binomial distribution	✓	https://github.com/BayraktarLab/cell2location	Python
DeconRNASeq [3]	Regression-based model; non-negative linear regression	×	https://www.bioconductor.org/packages/release/bioc/html/DeconRNASeq.html	R
DestVI [4]	Probability distribution-based model; negative binomial distribution	✓	https://docs.scvi-tools.org/en/stable/user_guide/models/destvi.html	Python
DWLS [5]	Regression-based model; dampened weighted linear regression	×	https://cran.r-project.org/web/packages/DWLS/index.html	R
SVR [5]	Regression-based model; nu-support vector regression	×	https://cran.r-project.org/web/packages/DWLS/index.html	R
MuSiC all gene [6]	Regression-based model; non-negative linear regression	×	https://github.com/xuranw/MuSiC	R
MuSiC weighted [6]	Regression-based model; weighted non-negative linear regression	×	https://github.com/xuranw/MuSiC	R
RCTD [7]	Probability distribution-based model; poisson distribution	✓	https://github.com/dmcable/spacexr	R
SCDC [8]	Regression-based model; non-negative linear regression and ensemble learning	×	https://github.com/meichendong/SCDC	R
SpatialDWLS [9]	Regression-based model; weighted non-negative linear regression	✓	https://github.com/RubD/Giotto	R
SPOTlight [10]	Regression-based model; non-negative matrix factorization	✓	https://github.com/MarcElosua/SPOTlight/tree/spotlight-0.1.7	R
STdeconvolve [11]	Latent dirichlet allocation	✓	https://github.com/JEFworks-Lab/STdeconvolve	R
Stereoscope [12]	Probability distribution-based model; negative binomial distribution	✓	https://github.com/almaan/stereoscope	Python

Table S2: Detailed cell type information in the mouse adult mouse cortical scRNA-seq dataset.

Cell type	Number of cells	Cell type	Number of cells
Astro	368	CR	7
Endo	94	L2/3IT	982
L4	1401	L5 IT	880
L5 PT	544	L6 CT	960
L6 IT	1872	L6b	358
Lamp5	1122	Macrophage	51
Meis2	45	NP	362
Oligo	91	Peri	32
Pvalb	1337	Serpinf1	27
SMC	55	Sncg	125
Sst	1741	Vip	1728
VLMC	67		

Table S3: Detailed cell type information in the PDAC-A scRNA-seq dataset.

Cell type	Number of cells	Cell type	Number of cells
Acinar cells	13	Cancer clone A	126
Cancer clone B	170	Ductal antigen presenting	287
Ductal centroacinar	529	Ductal high hypoxic	215
Ductal terminal	350	Endocrine cells	3
Endothelial cells	11	Fibroblasts	5
Macrophages A	21	Macrophages B	19
Mast cells	14	mDCs A	12
mDCs B	33	Monocytes	18
pDCs	13	RBCs	15
T cells and NK cells	40	Tuft cells	32

Notes: mDCs: myeloid dendritic cells; pDCs: plasmacytoid dendritic cells; RBCs; red blood cells.

Table S4: Detailed cell type information in the breast cancer scRNA-seq dataset.

Cell type	Number of cells	Cell type	Number of cells
B cells	162	CAFs (cancer-associated fibroblasts cells)	106
Epithelial	441	Endothelial	210
Myeloid	385	Plasmablasts	175
T cells	1473	PVL (Perivascular like cells)	72

3 Supplementary Text

3.1 Implementation of the individual deconvolution methods

3.1.1 CARD

CARD [1] uses a non-negative matrix factorization model to deconvolute SRT data based on the transcriptome signatures of cell type learned from reference scRNA-seq data. Compared with other deconvolution methods [2, 7, 10], CARD highlights that the neighboring locations (spots) on the tissue tend to contain similar cell type compositions. The pairwise distance between spots is calculated based on the 2-dimensional/3-dimensional spatial coordinate of the spots by Gaussian kernel. Then it is integrated into the deconvolution model to accommodate spatial correlation structure in cell type compositions across tissue locations. We obtain the CARD R package from `https://github.com/YingMa0107/CARD` and follow the guidelines on the CARD GitHub repository: `https://github.com/YingMa0107/CARD-Analysis`. All parameters are set with default values.

3.1.2 Cell2location

Cell2location [2] is built on a Bayesian model by decomposing spatially resolved gene expression profiles into signals from scRNA-seq data and technical effects such as platform effects, contaminating RNAs, and unexplained variants. It takes a hierarchical decomposition prior (factorization) to capture the similarity of spot patterns in cell type compositions. It is implemented in the scvi-tools framework on the Python platform (`https://github.com/BayraktarLab/cell2location`). We use the reticulate R package to develop an interface with the scvi-tools framework. There exist several hyper-parameters to be tunned in the model of Cell2location and two parameters, expected cell abundance per location and regularization of within-experiment variation in RNA detection sensitivity, are advised to be adjusted by the users in Cell2location [2]. As there is no true cell type abundance of spots in real application, it's difficult to select the optimal hyper-parameters. Hence, we take the default setting for the parameters advised by the guidelines on the Cell2location tutorial repository: `https://cell2location.readthedocs.io/en/latest/`.

3.1.3 DeconRNASeq

DeconRNASeq [3] is built upon a non-negative linear squares (nnls) regression model for the estimation of the proportions of known cell types in a sample of bulk RNA-seq data. In our implementation, the function *DeconRNASeq* in DeconRNASeq R package (`https://www.bioconductor.org/packages/release/bioc/html/DeconRNASeq.html`) is used for the deconvolution of SRT data. All parameters are set with default values.

3.1.4 DestVI

DestVI [4] is built on a Bayesian model for multi-resolution deconvolution of cell types in SRT data. Compared with other methods in our application, except for STdeconvolve, it enables to model of both discrete cell-type specific profiles and continuous within-cell-type latent variables through a conditional deep generative model. DestVI introduces two different latent variable models, scLVM and stLVM, to construct the reference scRNA-seq data and SRT data. For the scLVM model, DestVI first uses the negative binomial distribution to model the scRNA-seq data and takes the auto-encoding variational Bayes to optimize the likelihood of the estimation of

the parameters in the distribution. Then, stLVM adopts a weighted sum of the inferred single-cell negative binomial distribution incorporating the learned parameters from scLVM to fit the SRT data and uses maximum-a-posteriori (MAP) to estimate the cell-type abundance of spots. We follow the guidelines on the scvi-tools tutorial repository: `https://github.com/scverse/scvi-tutorials/blob/master/DestVI_tutorial.ipynb` and all parameters are set with default values. Similar to the application of cell2location, we use reticulate R package interface with Python for the implementation of DestVI in the R command.

3.1.5 DWLS and SVR

DWLS [5] is a computational tool for bulk RNA-seq deconvolution. The method adopts a weighted least square approach to infer the relative abundance of cell types, for which cell types are defined by reference scRNA-seq data. Besides, the authors also propose another deconvolution method, v-support vector regression (SVR) in their original paper. Both DWLS and SVR are included in our application. We obtain the code of DWLS and SVR from `https://cran.r-project.org/web/packages/DWLS/index.html` and follow the guidelines on the DWLS GitHub repository: `https://github.com/dtsoucas/DWLS`. DWLS includes an internal marker genes selection step by MAST [13] or SeuratBimod [14] for the construction of the signature matrix. In our application, we find this step takes long time consumption for scRNA-seq data with a large number of cell types or genes [15]. Hence, we take the Gini method for the selection of marker genes, which is also used in SpatialDWLS [9], an extended version of DWLS. In addition, the foreach R package is used for the parallel computation of the DWLS and SVR.

3.1.6 MuSiC

MuSiC [6] is a well-established computational method to deconvolute bulk RNA-seq data on multi-subject single-cell expression reference. It adopts a weighted nnls regression framework to infer cell type compositions. Notably, MuSiC incorporates the multi-subject scRNA-seq reference for deconvolution by assigning appropriate weights of cross-subject and cross-cell consistency genes. We obtain the Music R package from `https://github.com/xuranw/MuSiC` and follow the guidelines on `https://xuranw.github.io/MuSiC/articles/MuSiC.html`. All parameters are set with default values. Specifically, MuSiC provides two ways, "weighting gene" and "non-weighting genes", for deconvolution. We include all of them in this study and refer to them as "Music weighted" and "Music all gene", respectively.

3.1.7 RCTD

RCTD [7] leverages cell type profiles learned from scRNA-seq data and takes supervised learning to decompose cell type mixtures for SRT data. RCTD enables the correction of differences between the sequencing platforms, scRNA-seq and ST technology, for accurately mapping cell type populations on spots. A stepwise approach is adopted for the estimation of model parameters. The procedure of RCTD is easily accessible and we follow the guidelines on the RCTD GitHub repository: `https://raw.githack.com/dmcable/spacexr/master/vignettes/spatial-transcriptomics.html`. We set $doublet\_model = \text{"}full\text{"}$ in R function *run.RCTD*, which allows multiple cell types within a spot.

3.1.8 SCDC

SCDC [8] is designed for deconvoluting the bulk RNA-seq data by the cell-type specific gene expression profiles from multiple scRNA-seq reference datasets. By borrowing strengths from multiple references, SCDC proposes an ensemble learning-based deconvolution method to integrate deconvolution results from different scRNA-seq datasets sequenced in different laboratories and at different times. The function *SCDC_prop* in the SCDC package (`https://github.com/meichendong/SCDC`) is applied to deconvolute the cell type abundances for SRT data.

3.1.9 SpatialDWLS

As an extension of DWLS, SpatialDWLS [9] is designed for SRT data. Due to the fact that each spot contains much smaller cells than a bulk sample, SpatialDWLS first infer cell types that are likely to be presented at each spot by cell type enrichment analysis. Then, DWLS is applied to infer the fraction of selected cell types across spots. We follow the guidelines on the SpatialDWLS GitHub repository: `https://github.com/rdong08/spatialDWLS_dataset/tree/main/codes`. All parameters are set to default values.

3.1.10 SPOTlight

SPOTlight [10] is built on nonnegative matrix factorization and nnls for SRT data. Given the corresponding annotated reference scRNA-seq data, SPOTlight identifies the cell type-specific topic profiles, which enables determining the cell states and subpopulations for the deconvolution of SRT data. In our application, we first use the function *SCTransform* and *FindAllMaerkers* in the Seurat package to normalize the raw count scRNA-seq and to select maker genes for the pre-defined cell types. Then, the function *downsample_se_obj* and *train_nmf* in the SPOTlight package is applied for selecting represented cells and genes for each cell type and training the nonnegative matrix factorization model, respectively. Finally, we use the functions *topic_profile_per_cluster_nmf* and *mixture_deconvolution_nmf* in the SPOTlight package for deconvoluting SRT data and obtaining the cell type abundance in each spot. We take the default setting for the functions in the SPOTlight package (`https://github.com/MarcElosua/SPOTlight`).

3.1.11 Stereoscope

Stereoscope [12] is a probabilistic model-based method for SRT data. It first uses the negative binomial (NB) distribution to model the reference scRNA-seq data with pre-defined cell types. After training the reference model, it adopts another NB distribution incorporating the learned parameters from the reference model to fit the SRT data and infer the cell type abundance within each spot. Notably, Stereoscope utilizes the approximate variational inference to estimate the model's parameters and can be rescaled to large reference scRNA-seq data with more than one million cells. We follow the guidelines on the scvi-tools tutorial repository: `https://docs.scvi-tools.org/en/stable/tutorials/notebooks/stereoscope_heart_LV_tutorial.html`. All parameters are set with default values. In our experiment, we find that it is important to select highly variable genes (HVGs) for the scRNA-seq data before training the reference model. We select 5000 HVGs by Seurat.v3 [16] advised by the tutorial of Stereoscope published on `https://github.com/almaan/stereoscope`. Also, users could change the number of selected HVGs by setting the parameter *Stereoscope.HVG_num* of *EnDecon_individual_methods* in EnDecon R package. Since Stereoscope is built on Python, we use the reticulate R package interface with Python to implement it in the R command.

3.1.12 STdeconvolve

STdeconvolve [11] is built upon latent Dirichlet allocation (LDA) for the deconvolution of SRT data. By the advantage of the LDA model, compared with other methods, STdeconvolve enables to identify putative transcriptional profiles for each cell type and estimates the cell types proportions within each spot without using external scRNA-seq reference. In our application, we first use the function *restrictCopus* in the STdeconvolve to filter non-information genes and select over-dispersion genes for the model. Then, the function *fitLDA* in the package is applied for the fitting of the LDA model. STdeconvolve provides a strategy for the selection of the optimal number of topics, called cell type in STdeconvolve, in the LDA model. In our experiments, we find that STdeconvolve tends to select a smaller number of topics than the number of cell types of reference scRNA-seq and some cell types can't be mapped to the topics in the next steps. Hence, in our application, we set the number of topics being the pre-defined number of cell types in the reference of scRNA-seq. Finally, the function *getCorrMtx* is performed for the annotation of the topics in the LDA model with the external scRNA-seq reference and we can obtain the final cell type abundance within spots. The STdeconvolve provides two methods, transcriptional correlations and gene set enrichment analysis, for the annotation of the topics. We choose the transcriptional correlations in our study, which are also used for the benchmarking of deconvolution methods [17]. All parameters are set to default values and we follow the well-documented tutorials of STdeconvolve on `https://jef.works/STdeconvolve/`.

3.2 Summary of EnDecon

The complete procedure of EnDecon is summarized in Algorithm 1.

Algorithm 1 Algorithm of EnDecon

- **Inputs:** SRT data $X^1 \in \mathbb{R}^{p_1 \times n_1}$ and corresponding spot location information $V \in \mathbb{R}^{n_1 \times 2}$, scRNA-seq data $X^2 \in \mathbb{R}^{p_2 \times n_2}$, where the rows represent genes and the columns represent spots (or cells), and corresponding cell label vector $Y \in \mathbb{R}^{n_2}$ of the reference scRNA-seq data.

- **Output:** Ensemble deconvolution result H and weights $\{\omega_m\}$.

1. Run the base deconvolution methods to get multiple base deconvolution results $\{H^{(m)}\}$, for $m = 1, \ldots, M$.
2. **Initialization:** Set $w_1 = \cdots = w_M = \frac{1}{M}$, and λ by Equation (5) in the main text, and initialize $H = \frac{1}{M} \sum_{m=1}^{M} H^{(m)}$.
3. **While** not converged do
4. Update H by solving Equation (2) in the main text.
5. Update ω_m according to Equation (4) in the main text.
6. **End while**

3.3 Simulation data analysis

3.3.1 Generation of simulated SRT data

To test the performance of different methods, we design simulated SRT data consisting of mixtures of cells with predefined cell type compositions. Each spot consists of 2 to 10 different cells from the scRNA-seq dataset, and the combination of their expression levels is considered as the expression level at that spot [10]. To better mimic biological capture locations, we randomly downsample composed cells to 20,000 read counts if the read counts of the generated spot are up to 25,000. The proportions of the mixed cell types in each spot are served as the ground truth of cell type proportions information of generated data [1, 10]. Here, we generate simulation data in three different scenarios, which include spot-based gene expression data and corresponding cell type components within spots. Details are as follows:

Scenario 1: The SRT data and scRNA-seq dataset are generated from the same technology to examine the accuracy of EnDecon on cell type deconvolution. Following [1, 2], we divide the scRNA-seq dataset equally into two groups: one group is used to generate spot-based gene expression data to mimic the outcome of gene expression dataset from STR, and the other one is considered as the reference scRNA-seq dataset with annotated cell type labels. To test the generalizability of the proposed EnDecon, we generate simulated SRT data based on scRNA-seq data from two different tissues, i.e., the pancreas tissue and ovarian cancer tissue. In detail, we first select a publicly available human pancreas dataset from scRNA-seq protocol inDrop (named Baron), consisting of 7,742 cells and 6 common cell types (acinar, beta, delta, ductal, alpha and gamma) [18]. To explore the tumor microenvironment, we also generate simulated SRT data generated from ovarian cancer scRNA-seq data. We collect scRNA-seq data generated by 10x Genomic (number: OV_EMTAB8107) from link `http://tisch.comp-genomics.org/`. Since this dataset contains too many cells, we stratified downsampling the sample according to cell types from 3790 cells annotated with eight cell types (B (192), CD8T (427), Endothelial (259), Fibroblasts (981), Malignant (889), MonoMacro (569), Myofibroblasts (309), and Plasma (164)). For both datasets, we generate SRT data with 175 spots. To mimic the actual spots coordinates as much as possible, spot coordinates are set according to scenario 3.

Scenario 2: In this scenario, to demonstrate the robustness of our model on predicted cell type compositions, we mimic the case that the SRT and scRNA-seq data are generated from two different technologies on the same tissue. We use the same group scRNA-seq dataset of scenario 1 to generate 175 spots for the spot-based SRT data, and the scRNA-seq data from other technique serves as reference data. For the human pancreas, there are multiple scRNA-seq data generated by different technologies (`https://hemberg-lab.github.io/scRNA.seq.datasets/human/pancreas/`). To evaluate the deconvolution results, we consider scRNA-seq data as reference data if that contains matched six cell types in scenario 1. The scRNA-seq datasets generated from other three techniques (e.g., Muraro: CEL-Seq2 [19], Segerstolpe: SMART-Seq2 [20], and Wang: SMARTer [21]) are regarded as reference cell type-specific gene expression data in this work.

Scenario 3: SRT data is simulated based on the real single-cell resolution spatial transcriptomics data in this scenario. We select a public STARmap dataset, which contains the expression levels of 981 genes in 973 cells from the mouse visual cortex at the single-cell resolution and is refined with six neocortical layers (Figure S6) [22]. To generate coarse-grained SRT data from single-cell resolution data, we define one spot-based region by the size of the grid and aggregate the gene expression level that fall into each spot [9]. After gridding, a total of 175 spots are simulated and each spot covers 1-13 cells. As the cell type labels of the selected cells are known, the resulting cell type compositions of each spot can be used as the ground truth for evaluation. The center of the grids is served as the coordinates of the corresponding generated spots.

For deconvolution, a mouse primary visual cortex (VISp) scRNA-seq dataset from Smart-seq protocol is regarded as the reference [23].

3.3.2 Effect of sample sizes of the reference scRNA-seq data on the performance of deconvolution

To explore how the sample sizes of the reference scRNA-seq data affect the results, we evaluate the performance of cell type deconvolution methods with different reference sample sizes. For this goal, the Baron dataset used in scenario 1 is considered. For a given reference scRNA-seq data, we use a stratified subsampling approach to divide cells into subpopulations according to their types and sample each subpopulation independently to generate sub-reference datasets. Four sub-reference datasets are generated with down-sampling ratios of 0.2, 0.4, 0.6, and 0.8. The details of reference sample sizes are listed in Table S5. Thus, we generate four different sample sizes of reference scRNA-seq data against the whole reference scRNA-seq data. We run each deconvolution method with these sub-reference datasets and compare the results with changes in downsampling rate.

Under PCC, RMSE, and JSD metrics, we find all methods (except DestVI) are not very sensitive to the sample sizes of the reference scRNA-seq datasets (Figure S23). This may be because most deconvolution methods only use reference scRNA-seq data to estimate the gene expression profiles of each cell type. If the estimated cell type-specific gene expression profiles are able to capture the distribution of the corresponding cell types and distinguish different cell types, the deconvolution results of these methods may not change much. For DestVI, it adopts amortized variational inference with deep neural networks to learn the cell-type-specific representations of cell states on reference datasets, which rely on the number of cells. Therefore, we observe that its performance increases with the number of cells in the reference dataset. In summary, our method and most individual methods do not depend heavily on the number of cells in the reference scRNA-seq dataset. Therefore, in our opinion, reference scRNA-seq datasets that contain a certain number of cells, are low in noise, and have small technical and biological differences from the SRT data may all be appropriate in practice.

Table S5: **Detailed cell type information of the reference scRNA-seq dataset in downsampling experiments.**

downsampling ratio	Number of cells per cell type					
	acinar	beta	delta	ductal	alpha	gamma
1	479	1163	1263	301	539	128
0.8	384	931	1011	241	432	104
0.6	288	699	759	181	324	78
0.4	192	467	507	121	216	52
0.2	96	234	254	61	108	26

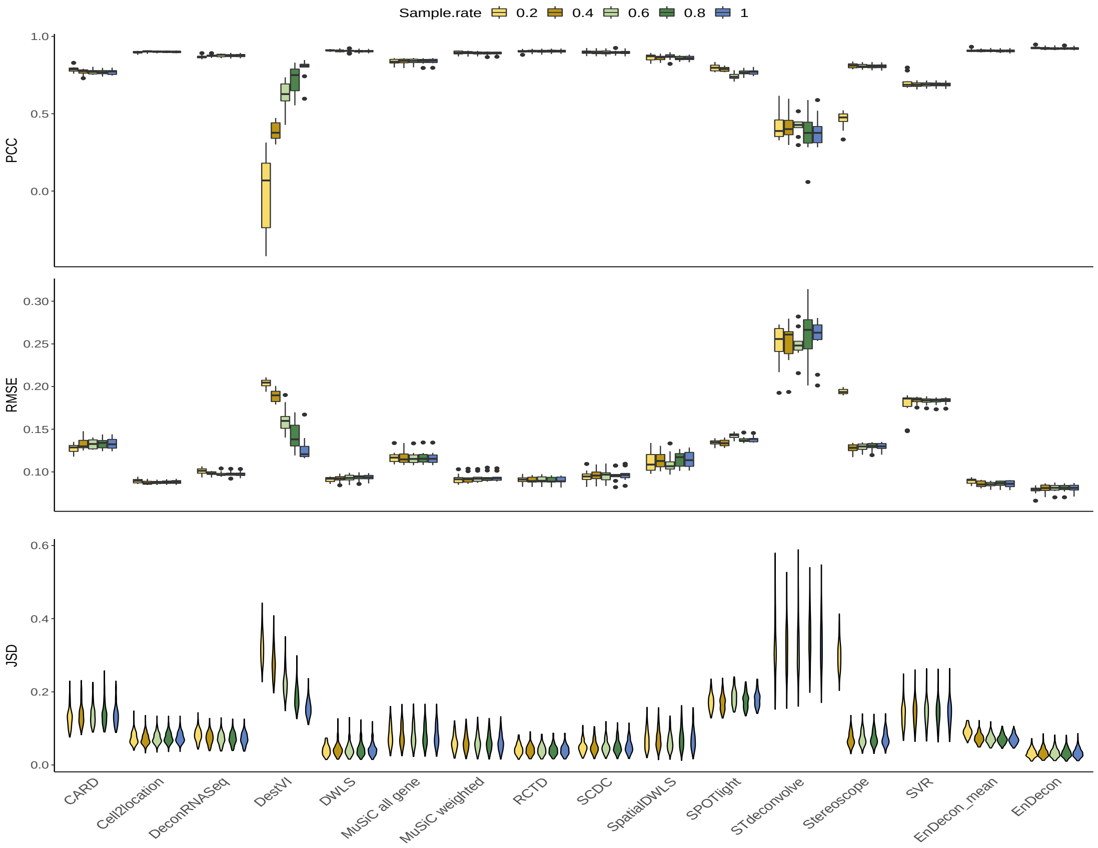

Figure S23: Performance of the compared methods on simulation data with different sample sizes of reference scRNA-seq datasets. The subfigures correspond to different evaluation metrics. In the subfigure, each color represents a downsampling ratio (0.2, 0.4, 0.6, 0.8, and 1) on the reference datasets. The results are averaged over 10 random generations of the data.

3.3.3 Running time comparison

For a computational method, the accuracy is important, but the running time also needs to be considered. Therefore, we also report the computational time requirement for the deconvolution methods. To obtain the running time, we run the deconvolution methods on a workstation with Intel core i7-10700 CPU (2.90GHz $\times$ 16), 64 RAM and RTX 3080 GPU. Figure S24 presents the running times of the 14 individual methods as well as our ensemble process on the six datasets across the three scenarios in the simulation experiments. All individual deconvolution methods can be finished in less than 50 minutes on a given dataset. Cell2location, DestVI, DWLS, and Stereoscope require more times than other methods. Note that after running the individual methods, EnDecon can integrate the base results in a short time. In addition, we also provide an overview of the deconvolution methods in term of PCC, 1-RMSE, and 1-JSD, and running time on all simulated datasets (Figure S25) for the users to select appropriate individual deconvolution methods for integration.

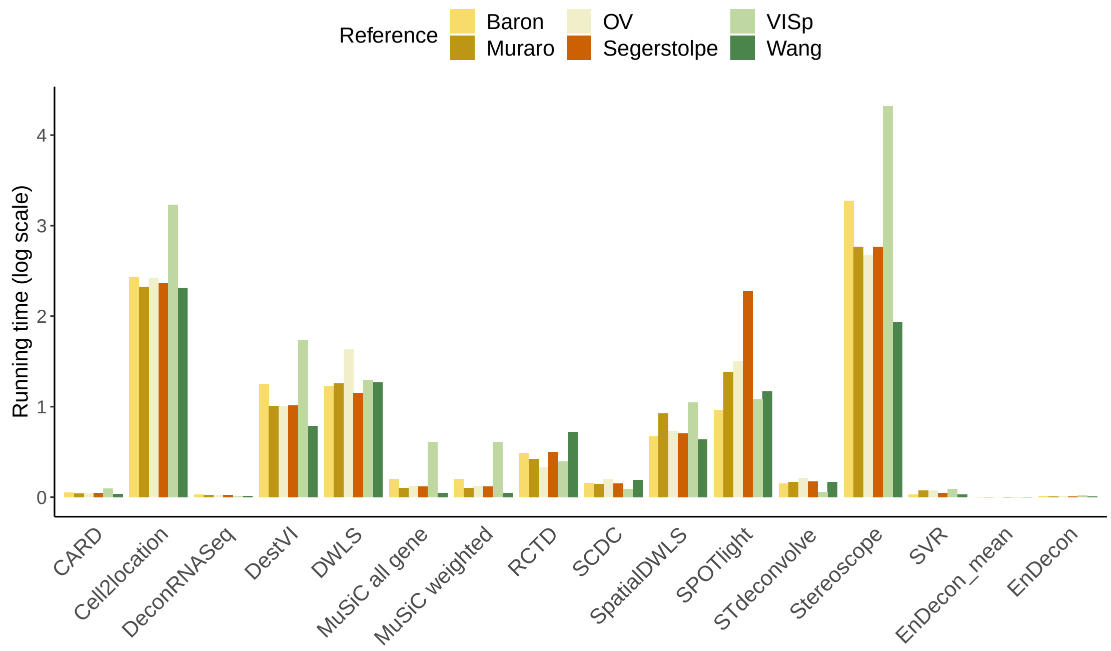

Figure S24: Running times of different deconvolution methods on the simulation data in scenarios 1 and 2. The x-axis is the deconvolution methods, and the y-axis is the log scale of running times in minutes of each method. Each color represents reference scRNA-seq data for the deconvolution. Notice that, for our EnDecon, only the time used for integrating deconvolution results from base methods is presented.

3.4 Real SRT data analysis

3.4.1 Collecting real SRT data and reference scRNA-seq dataset

We apply EnDecon to four published SRT data that include two from the ST protocol and two from the 10x Visium protocol. We use corresponding scRNA-seq datasets as references.

Adult Mouse Brain. We first analyze an adult mouse brain SRT data of coronal section 2 downloaded from the 10x Genomics website: (`https://www.10xgenomics.com/resources/datasets/adult-mouse-brain-section-2-coronal-stains-dapi-anti-gfap-anti-neu-n-1-standard-1-1-0`). To do cell type deconvolution, the reference scRNAseq dataset (GSE71585) is generated from adult mouse cortical cell taxonomy with the SMART-Seq2 protocol provided by the Allen institute, which contains ∼14,000 cells and 23 annotated cell types [24]. In this work, we filter out genes expressed on less than 5% cells (spots) and cells with less than 100 total read counts. 11,764 common genes are expressed in 2,804 spots in the adult mouse brain SRT data, and these expressed genes are present in 8,412 cells (Table S2).

For this SRT data, the 10x Genomics platform provides SRT data along with cell type-informative images, e.g., immunofluorescence (IF) staining images for two cell type-specific marker proteins (glial cells marker protein: GFAP, and neuron cells marker protein: RBFOX3). The staining IF images are collected from the backside of tissue sections affixed to spatial transcriptomics capture slides. Following [25], we use spot-level intensities which are the average pixel intensity of each of these two markers in all image pixels overlapping each capture spot

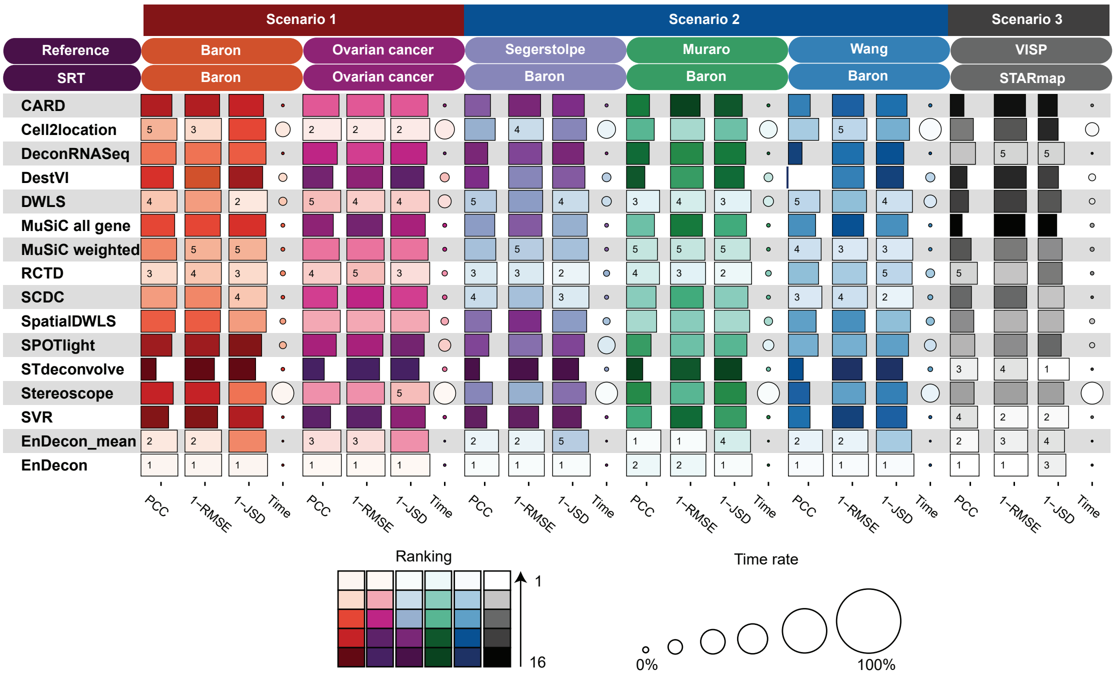

Figure S25: Overview of deconvolution methods by PCC, 1-RMSE, 1-JSD and running times for the six datasets across three scenarios in the simulation experiments. The lightness of colors filled in each box is proportional to the rank of each method. The time consumption is proportional to the size of the circle. Notice that we only label the first top five methods in experiments of each metric.

on the Visium slide, as the ground truth of cell type proportions. The calculated cell type proportions for glial and neuron cells are available from the link: `https://osf.io/u79fc/`.

Human pancreatic ductal adenocarcinoma (PDAC). We consider the human PDAC data from ST technology, consisting of four main annotated tissue regions (cancer, pancreatic, ductal and stroma regions) annotated by histologists based on $H\&E$ staining image [26]. For deconvolution, we use a matched scRNA-seq dataset on the same tissue of the same individual obtained through inDrop (denoted as PDAC-A) [26]. The PDAC datasets are downloaded from the Gene Expression Omnibus (GEO) website (GSE 111672), which provides both SRT data and the corresponding scRNA-seq dataset on the same PDAC tissue. In this analysis, the genes expressed on less than 1% of cells (spots) and the cells that have less than 100 total read counts are filtered. The filtering step leads to SRT data consisting of 9,923 common genes expressed in 427 spots. It also leads to a scRNA-seq dataset including 9,923 common genes, 1,914 cells and 20 annotated major clusters (Table S3).

Human breast cancer. The mouse brain cortex is a complex tissue that comprises a complex mixture of cell types that present well-defined structures with location-specific types. We also focus on human breast cancer data from ST technology (Section D1 of patient D), and use breast cancer scRNA-seq data from 10x Chromium protocol as reference [27, 28]. The SRT data consists of three annotated regions (connective tissue (CT), immune infiltrate (II) and invasive cancer (IC) regions) and one undetermined (UN) region, annotated by a pathologist based on the morphology of the associated $H\&E$ staining [27]. The data are available at the Zenodo data

repository (`https://doi.org/10.5281/zenodo.4739739`). Following the original paper, we focus on the scRNA-seq dataset for the CID3921 HER2-positive patient, which is available on the GEO website under accession number ID GSM5354515 [27, 28]. Based on the filtering criterion mentioned above, a final set of 11,920 genes and 306 spots for SRT data and 11,920 genes and 3,024 cells for scRNA-seq data (Table S4) are induced.

Mouse brain cortex. The mouse brain cortex is a complex tissue that comprises a complex mixture of cell types that present well-defined structures with location-specific types. To explore the cell types' spatial distributions, we select SRT data of sagittal mouse brain slice (anterior slice) generated using the Visium v1 chemistry from the 10x Genomics website. The mouse cortex region containing 1,074 spots and 31,053 genes is analyzed in this work. Here, we use the same reference scRNA-seq data for the adult mouse brain tissue from the cornel section as a reference dataset. After removing none expressed genes and low-quality cells by the filtering criterion mentioned above, SRT data includes 13,456 shared genes and 1,074 spots, and the reference scRNA-seq dataset consists of 14,242 cells annotated by 23 different cell types (Table S2).

3.4.2 Performance evaluation on real SRT data

Cell type colocalization analysis. The cell type colocalization map is quantified by computing the spot-wise PCC based on the predicted cell type proportions [12, 27]. A positive correlation between two cell types indicates colocalization in spatial tissue, and the degree of colocalization is proportional to the correlation value. In contrast, a negative correlation indicates the opposite. More specifically, for each pair of cell types (k and k'), PCC is computed,

$$PCC(k,k') = \frac{\sum_{i=1}^{n}(h_{ik}-\bar{h}_k)(h_{ik'}-\bar{h}_{k'})}{\sqrt{\sum_{i=1}^{n}(h_{ik}-\bar{h}_k)^2}\sqrt{\sum_{i=1}^{n}(h_{ik'}-\bar{h}_{k'})^2}},$$

where h_{ik} represents the proportion of cell type k in spot i, and $\bar{h}_k$ represents the arithmetic mean of the proportion of cell types k.

Region-based cell type enrichment analysis. To assess the enrichment, or depletion, of the predicted cell type proportions in relation to annotated spatial regions [27], we first calculate the average of the proportion of spots containing each cell type in each segmented region, referred as the true average. We then randomly permute the predicted cell type proportions vector 10,000 times for each cell type, while maintaining the original spots annotation information. The average proportions of each annotated region are calculated and determined by each permutation, referred as permuted average. The enrichment score of each cell type in each region is taken as the mean value of the scaled difference between the true average and permuted average.

3.4.3 Mouse brain cortex deconvolution

The fourth dataset we examine is the mouse brain cortex dataset from 10x Visium, using the same reference scRNA-seq dataset of the first real SRT data as a reference dataset (Table S2). The mouse brain cortex presents well-defined structures, consisting of six layers from inside to out (Figure S26a). After deconvoluting, the cell type compositions inferred by EnDecon accurately depict such expected structures of mouse brain anatomy (Figure S26b). The compared methods "MuSiC all gene" and Stereoscope are unable to distinguish these six layers from each

other, and CARD shows a blurry boundary between layers (Figure S27). The dominant cell types within each spot are displayed from the outside layer to the inner: Astro, L2/3 IT, L4, L5 IT, L6 CT, L6 IT, L6b, and Oligo, respectively (Figure S26c). A closer examination of the spatial distribution of dominant cell types on their known structures confirms the high accuracy of the EnDecon predictions. In contrast, the compared methods, such as MuSiC, SCDC, SPOTlight, and Stereoscope, are unable to reveal clear spatial distribution patterns in these six layers (Figure S28).

We perform a concordance analysis of the spatial distribution of eight dominant cell types by EnDecon and their corresponding canonical cell type marker genes obtained from [24] (Figure S26d). Two non-neurons (Oligo and Astro) are located at the innermost and outermost parts of the tissue, respectively, which are consistent with the spatial expression patterns of their corresponding marker genes (Serpinbla and Aqp4). Two layer-specific neuronal subtypes L5 PT cells and L5 IT cells are located in the same layer 5 with different spatial distribution patterns, which align with the expression patterns of related marker genes (Hsd11b1 and Chrna8). These results illustrate the ability of EnDecon to discriminate between similar cell types within complex tissues.

Most of the total 23 annotated cell types are located in distinct regions and show clear spatial distribution patterns (Figure S29). We also observe that most cell types estimated by EnDecon appear to have spatial colocalization patterns (Figure S26e). Non-neuronal cells (such as Endo, Astro, Macrophage, SMC, and VLMC) present strong colocalization modules. The dominant cell types in the neighborhood layer also show strong positive correlations, such as L6 CT and L6 IT, L2/3 and L4.

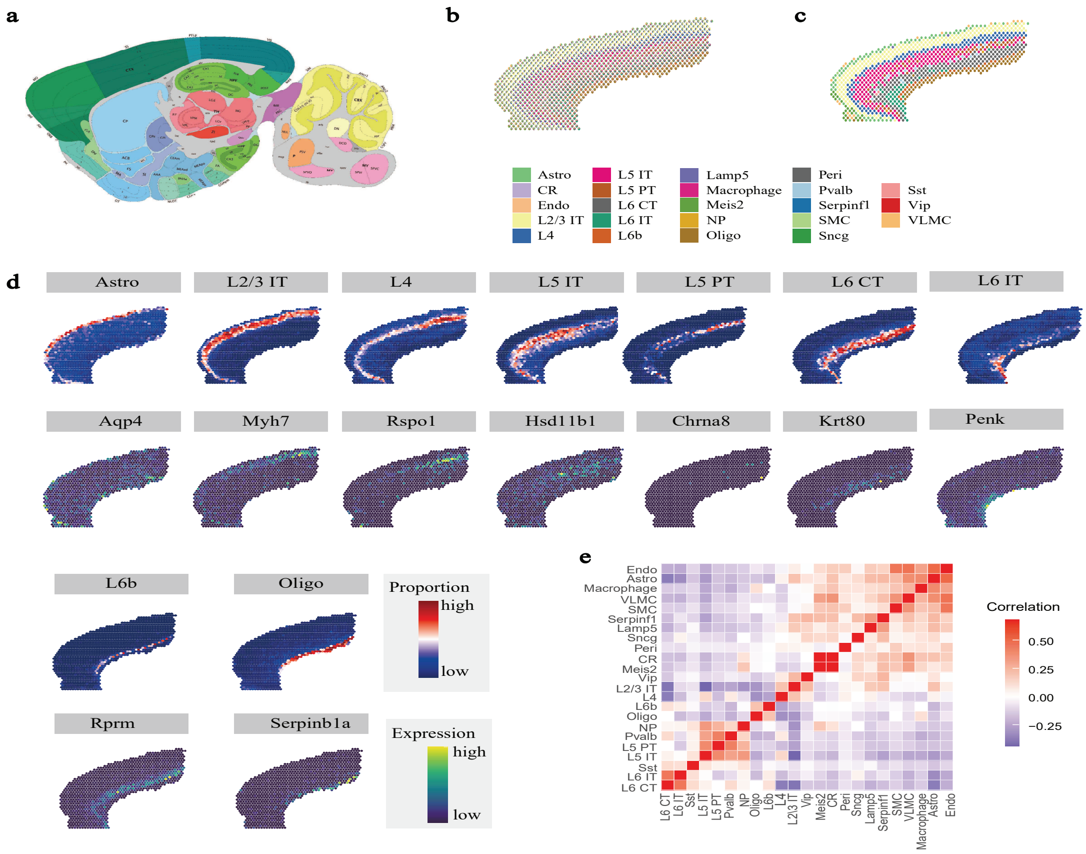

Figure S26: Analysis of the mouse brain cortex data. (a) Allen Brain Institute reference atlas diagram of the mouse cortex with well-defined six neocortical layers. (b) Visualization of deconvolution result by EnDecon. A spatial scatter pie chart displays cell type compositions predicted by EnDecon. Each scatter represents a spot in SRT data. (c) Visualization of dominant cell types inferred by EnDecon. A spatial scatter pie chart displays the spatial distribution of dominant cell types on each spot. (d) Top, the abundances of dominant cell types estimated by EnDecon are visualized on each spatial location. Bottom, the expression level of the corresponding canonical cell type marker genes is displayed. (e) Spatial colocalization map of predicted cell type by EnDecon. The correlation plot shows a different correlation across diverse cell types.

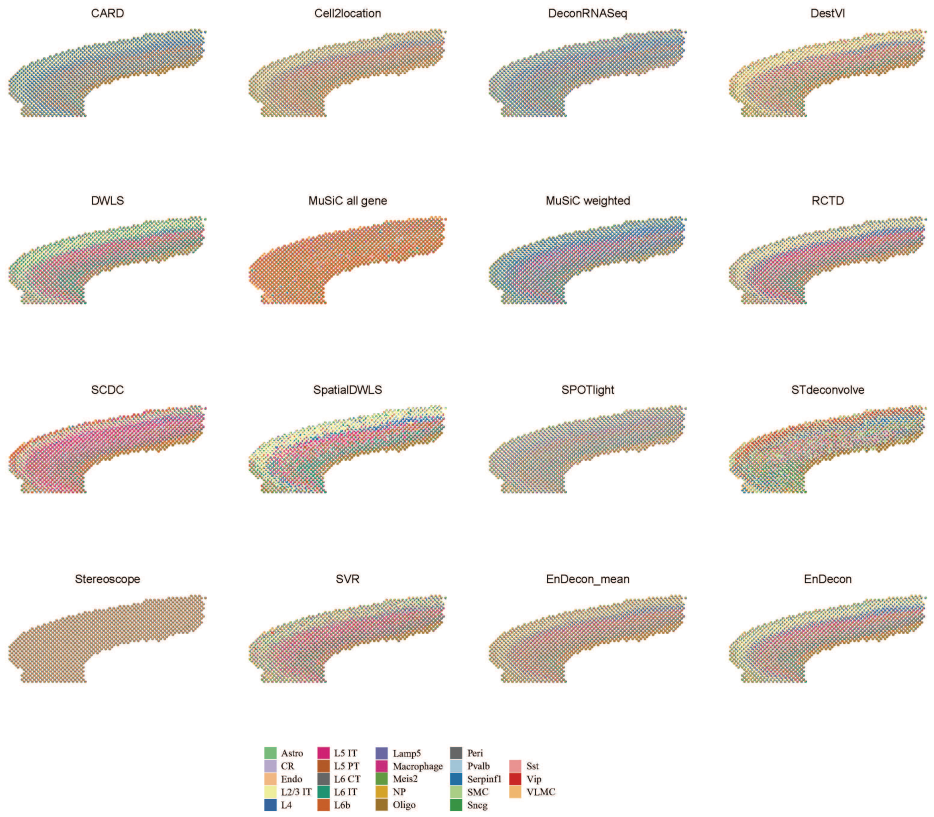

Figure S27: Visualization of deconvolution results inferred by the base deconvolution methods, EnDecon_mean and EnDecon from mouse brain cortex SRT data. The spatial scatter pie chart displays cell type compositions predicted by the deconvolution method and the scatter represents a spot in SRT data.

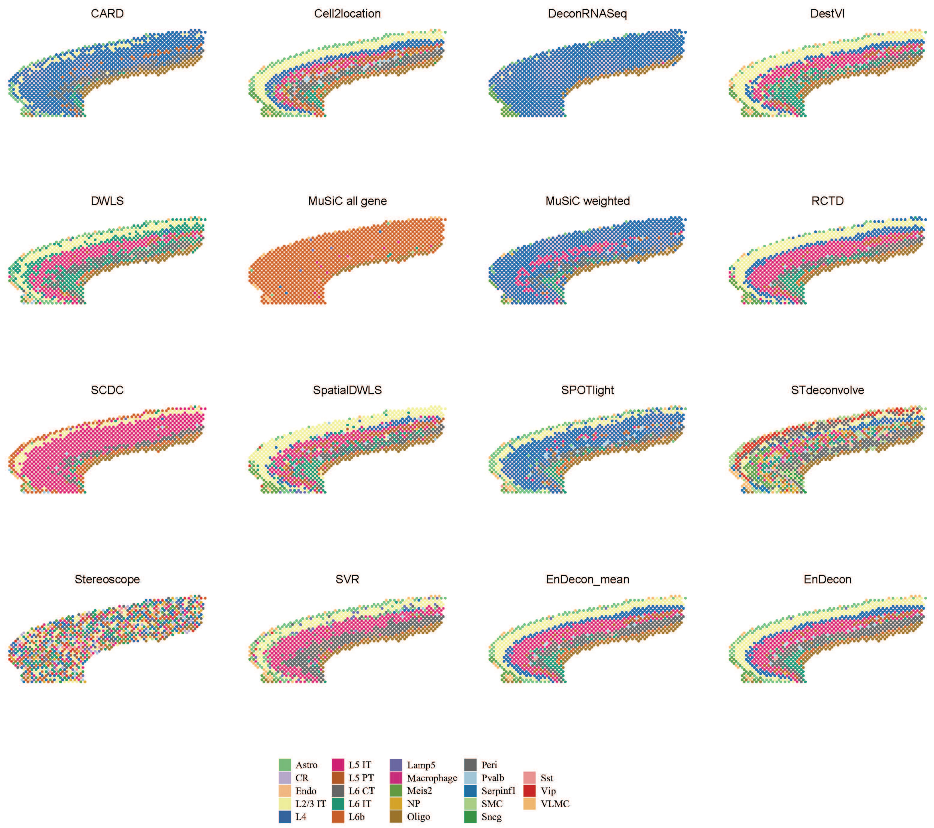

Figure S28: Visualization of dominant cell types inferred by the base deconvolution methods, EnDecon_mean and EnDecon from mouse brain cortex SRT data. The spatial scatter chart displays the spatial distribution of dominant cell types on spot by the deconvolution method.

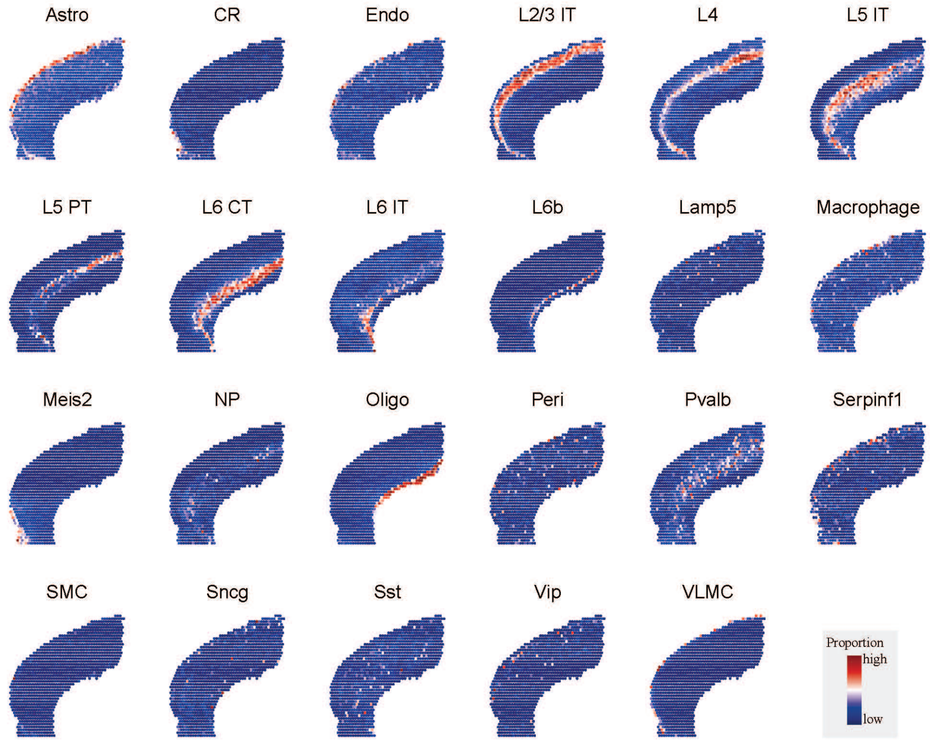

Figure S29: Visualization of cell type proportions predicted by EnDecon from mouse brain cortex data. The spatial scatter plot displays the spatial distribution of each cell type.

References

[1] Ying Ma et al. Spatially informed cell-type deconvolution for spatial transcriptomics. *Nat. Biotechnol*, pages 1–11, 2022.

[2] Vitalii Kleshchevnikov et al. Cell2location maps fine-grained cell types in spatial transcriptomics. *Nat. Biotechnol*, pages 1–11, 2022.

[3] Ting Gong et al. Deconrnaseq: a statistical framework for deconvolution of heterogeneous tissue samples based on mrna-seq data. *Bioinformatics*, 29(8):1083–1085, 2013.

[4] Romain Lopez et al. Destvi identifies continuums of cell types in spatial transcriptomics data. *Nat. Biotechnol*, pages 1–10, 2022.

[5] Daphne Tsoucas et al. Accurate estimation of cell-type composition from gene expression data. *Nat. Commun*, 10(1):1–9, 2019.

[6] Xuran Wang et al. Bulk tissue cell type deconvolution with multi-subject single-cell expression reference. *Nat. Commun*, 10(1):1–9, 2019.

[7] Dylan M Cable et al. Robust decomposition of cell type mixtures in spatial transcriptomics. *Nat. Biotechnol*, pages 1–10, 2021.

[8] Meichen Dong et al. Scdc: bulk gene expression deconvolution by multiple single-cell rna sequencing references. *Brief. Bioinformatics*, 22(1):416–427, 2021.

[9] Rui Dong et al. Spatialdwls: accurate deconvolution of spatial transcriptomic data. *Genome Biol*, 22(1):1–10, 2021.

[10] Marc Elosua-Bayes et al. Spotlight: seeded nmf regression to deconvolute spatial transcriptomics spots with single-cell transcriptomes. *Nucleic Acids Res*, 49(9):e50–e50, 2021.

[11] Brendan F Miller et al. Reference-free cell type deconvolution of multi-cellular pixel-resolution spatially resolved transcriptomics data. *Nat. Commun*, 13(1):1–13, 2022.

[12] Alma Andersson et al. Single-cell and spatial transcriptomics enables probabilistic inference of cell type topography. *Commun. Biol*, 3(1):1–8, 2020.

[13] Greg Finak et al. Mast: a flexible statistical framework for assessing transcriptional changes and characterizing heterogeneity in single-cell rna sequencing data. *Genome Biol*, 16(1):1–13, 2015.

[14] Rahul Satija et al. Spatial reconstruction of single-cell gene expression data. *Nat. Biotechnol*, 33(5):495–502, 2015.

[15] Hui-Sheng Li et al. scdea: differential expression analysis in single-cell rna-sequencing data via ensemble learning. *Brief. Bioinformatics*, 23(1):bbab402, 2022.

[16] Tim Stuart et al. Comprehensive integration of single-cell data. *Cell*, 177(7):1888–1902, 2019.

[17] Jiawen Chen et al. A comprehensive comparison on cell-type composition inference for spatial transcriptomics data. *Brief. Bioinform.*, 23(4):bbac245, 2022.

[18] Maayan Baron et al. A single-cell transcriptomic map of the human and mouse pancreas reveals inter-and intra-cell population structure. *Cell Syst*, 3(4):346–360, 2016.

[19] Mauro J Muraro et al. A single-cell transcriptome atlas of the human pancreas. *Cell syst*, 3(4):385–394, 2016.

[20] Åsa Segerstolpe et al. Single-cell transcriptome profiling of human pancreatic islets in health and type 2 diabetes. *Cell Metab*, 24(4):593–607, 2016.

[21] Yue J Wang et al. Single-cell transcriptomics of the human endocrine pancreas. *Diabetes*, 65(10):3028–3038, 2016.

[22] Xiao Wang et al. Three-dimensional intact-tissue sequencing of single-cell transcriptional states. *Science*, 361(6400):eaat5691, 2018.

[23] Bosiljka Tasic et al. Shared and distinct transcriptomic cell types across neocortical areas. *Nature*, 563(7729):72–78, 2018.

[24] Bosiljka Tasic et al. Adult mouse cortical cell taxonomy revealed by single cell transcriptomics. *Nat. Neurosci*, 19(2):335–346, 2016.

[25] Asif Zubair et al. Cell type identification in spatial transcriptomics data can be improved by leveraging cell-type-informative paired tissue images using a bayesian probabilistic model. *Nucleic Acids Res*, 2022.

[26] Reuben Moncada et al. Integrating microarray-based spatial transcriptomics and single-cell rna-seq reveals tissue architecture in pancreatic ductal adenocarcinomas. *Nat. Biotechnol*, 38(3):333–342, 2020.

[27] Alma Andersson et al. Spatial deconvolution of her2-positive breast cancer delineates tumor-associated cell type interactions. *Nat. Commun*, 12(1):1–14, 2021.

[28] Sunny Z Wu et al. A single-cell and spatially resolved atlas of human breast cancers. *Nat. Genet.*, 53(9):1334–1347, 2021.
